# Portuguese Version of the MISSCARE Survey-Patient: A Cross-Cultural Validation Study

**DOI:** 10.3390/nursrep16090312

**Published:** 2026-09-01

**Authors:** Paulo Cruchinho, Gisela Teixeira, Pedro Lucas, Filomena Gaspar, María Dolores López-Franco

**Affiliations:** 1 Inter-University Doctoral Studies in Comprehensive Care and Health Services (UJA-UDL-UVIC), University of Jaén, 23071 Jaén, Spain; 2 Nursing Research Innovation and Development Centre of Lisbon (CIDNUR), School of Nursing, Universidade de Lisboa, 1600-190 Lisbon, Portugal; gteixeira@campus.enfermagem.ulisboa.pt (G.T.); prlucas@enfermagem.ulisboa.pt (P.L.); mfgaspar@enfermagem.ulisboa.pt (F.G.); 3 Nursing Administration Department, School of Nursing, Universidade de Lisboa, 1600-190 Lisbon, Portugal; 4 CTS-464 Nursing and Innovation in Healthcare, Department of Nursing, University of Jaén, 23071 Jaén, Spain; mlfranco@ujaen.es

**Keywords:** missed nursing care, health care rationing, patient-reported experience measures, patient safety, comprehensive health care, nursing administration research

## Abstract

**Background:** Missed Nursing Care is a comprehensive, multidimensional, and frequently occurring phenomenon in various clinical contexts in Portugal. **Objective**: This study aimed to translate, adapt, and cross-culturally validate the MISSCARE Survey-Patient for the Portuguese population and to analyze the quality of its measurement properties. **Methods**: An exploratory–descriptive, quantitative, cross-sectional validation study was conducted. The questionnaire underwent cross-cultural adaptation through translation, translation synthesis, and back-translation procedures, followed by harmonization by an expert committee, pre-testing, and field testing. **Results**: Ten hospitalized persons and ten nurses participated in the pre-test as experts, while 135 hospitalized persons were included in the field testing. In the pre-testing, excellent content validity indices were obtained for clarity (S-CVUI/Med of 0.98 and S-CVI/UA of 0.84) and cultural relevance (S-CVUI/Med and S-CVI/UA of 1.00) for the overall instrument. In the field testing, a final three-dimensional model (Basic Care, Communication, and Time to Response) was obtained, explaining 66.7% of the total variance. Evidence based on internal structure was supported by Structural Validity indices from the confirmatory factor analysis [X^2^(56) = 52.421, *p* < 0.001, χ^2^/df = 2.722; PCFI = 0.640 and CFI = 0.892]. Convergent Validity was demonstrated through a moderate correlation between the instrument and a question assessing satisfaction with care (r = −0.677; *p* < 0.001). For the overall instrument, Cronbach’s α was 0.818, indicating very good Internal Consistency, while values for the dimensions ranged between 0.766 and 0.902. Minimal Important Change exceeded the Smallest Detectable Change by seven points for the total instrument, suggesting good potential to detect meaningful changes. **Conclusions**: The MISSCARE Survey-Patient-PT demonstrates good measurement properties for assessing hospitalized patients’ perceptions of the occurrence of Missed Nursing Care in Portuguese health services.

## 1. Introduction

In recent decades, Patient Safety has become a phenomenon of great importance and increased concern worldwide. The World Health Organization (WHO) [1] defines Patient Safety as a set of activities organized in a healthcare environment that creates cultures, processes, procedures, behaviors, and technologies to consistently and sustainably reduce risks, decrease the occurrence of preventable harm, make errors less likely, and reduce the impact of harm when it occurs. In Portugal, the Direção Geral de Saúde (General Directorate of Health) [2] has been responsible for coordinating and implementing national Patient Safety plans for healthcare organizations and professionals. Currently, the National Patient Safety Plan 2021–2026 is in force, which aims to strengthen and encourage safety in the provision of healthcare, based on five pillars: (1) Safety Culture; (2) Leadership and Governance; (3) Communication; (4) Prevention and Management of Safety Incidents; and (5) Safe Practices in Safe Environments. Each of these pillars provides for a variable set of activities with significant involvement from nurses.

Nurses play an essential role in maintaining Patient Safety in healthcare organizations as they monitor hospitalized persons 24 h a day, seven days a week. However, due to the high complexity of hospitalized persons’ care needs and the shortage of nurses to meet those needs, ensuring Patient Safety can be a major challenge in nursing practice settings. In recent years, this challenge has led to successive reports in the Portuguese media regarding the growing number of requests from nurses to be exempted from responsibility for the safety and quality of the nursing care they provide. For example, in the first half of 2025, around 1000 requests were reported [3], and more recently, in just over one month, nearly 500 requests were recorded [4], reflecting the inability of nurses to safely meet the basic care needs of hospitalized persons. Globally, this phenomenon is known as Missed Nursing Care (MNC). 

## 2. Background

MNC is recognized as a comprehensive, multidimensional, and frequent phenomenon across different clinical contexts. Although it is a global phenomenon, it occurs in different ways depending on the characteristics of health organizations in each region and/or country. Studies reporting the occurrence of MNC communicated by nurses have indicated prevalence rates in various countries ranging from 97.5% to 30% [5,6,7,8,9,10]. In clinical settings, MNC is known to occur in emergency departments [10,11], intensive care [5,12,13], home care [14], mental and psychiatric health services [15], pediatric care units [16,17], neonatal units [18], stomatherapy services [19], oncology care units [20], medical units [21], maternal and obstetric health [22,23], surgical care units [24], post-anesthesia care units [25], and health community units [26]. MNC has also been identified in nursing practice environments that simultaneously function as learning contexts for nursing students [27,28].

Studies conducted on MNC have used inconsistent terminology to refer to the phenomenon of omitted nursing care. Terms used include Missed Nursing Care [29], Care Left Undone [30], Tasks Left Undone [31,32], Rationing of Nursing Care [33], Unmet Nursing Care Needs [34,35], Unfinished Nursing Care [36], and Failure to Maintain [37]. Currently, there is still no consensus among researchers regarding the use of a single universal term to define the phenomenon of care omission after more than two decades of research. In addition to terminological inconsistency, the various terms are difficult to translate, making international comparisons of research data difficult. Due to terminological inconsistency, some authors have adopted the term Care Rationed or Missed to refer to any type of nursing care that is needed but not provided [38]. Among all the terms used in the literature, Missed Nursing Care is the most widely adopted in international research. The recommended Portuguese translation is *Falta de Cuidados de Enfermagem* (Lack of Nursing Care) [39]. In this study, the term Missed Nursing Care was used because it defines the phenomenon under study more accurately, reducing ambiguity and narrowing its scope of application.

The care activities most frequently omitted by nurses in different countries and clinical settings have been reported mainly by nurses and less by hospitalized persons. A systematic review conducted by Sarpong et al. [40], which analyzed 45 studies on MNC in acute care settings, reported that the most frequently missed activity was ambulation, with an estimated prevalence of 46%, followed by mouthcare (36%), emotional support (33%), bathing (31%), and feeding (30%). An umbrella review conducted by Chaboyer et al. [29] identified the main categories of missed care as communication and information sharing, self-management, autonomy, and education, including care planning, discharge planning and decision-making, fundamental physical care, and emotional and psychological care, including spiritual support. In a study conducted by Connolly et al. [41], while nurses reported missing participation in interdisciplinary care conferences, mobilization, and oral care, hospitalized persons most frequently reported omissions in oral care, communication regarding their assigned nurse, and mobilization.

Scientific literature also reports the significant impacts of MNC at the organizational, professional, and patient levels. Organizational impacts include less/poorer quality of patient care [29,42], an increasing hospital length of stay [29], hospital readmissions [29,43], patient outcomes in the context of Patient Safety and quality of nursing care [44,45], decreased hospital credit [42], increased healthcare costs [42,46], and a decline in the social image of nursing [47]. At the nurse level, consequences include emotional responses, including guilt, frustration, and helplessness [48], moral distress [49,50], job burnout [51,52], nursing turnover [42,53], intention to leave [7,54], absenteeism [42], job dissatisfaction [7,23], and negative effects on the professional socialization of nursing students [55]. Finally, for hospitalized persons, the consequences of missed care in adult settings include increased mortality [56], adverse events [29,57], critical incidents [43], failure to maintain [57], dissatisfaction with care [58], and loss of trust in nurses [47]. 

Some authors have explored the occurrence of adverse events as a result of MNC. For example, Cho et al. [59], in a study conducted in 23 units of six hospitals in South Korea, reported that the most frequent adverse events associated with MNC were patient falls, skin lesions or pressure ulcers, healthcare-associated infections, medication administration errors, and intravenous infusion complications. These adverse events are commonly referred to as Nurse Sensitive Adverse Events or Nurse Sensitive Outcomes and are defined as adverse events that are sensitive to variations in nursing care and are strongly influenced by nurses’ actions [60]. Such events are classified as errors of omission, as opposed to errors of commission associated with performing certain actions incorrectly, as they are characterized by the failure to provide certain nursing care or by the same not being completed or being postponed by nurses [46]. Therefore, MNC is considered the key indicator of Patient Safety [61].

Despite limited intervention research aimed at reducing MNC, most reported interventions focus on changing or optimizing relevant care processes [38]. Longhini et al. [62] highlighted the role of Nurse Managers (NMs) in reducing MNC through nurse engagement, the redesign of nursing documentation, strengthening nursing leadership, and improving staff performance. A study conducted by Janatolmakan et al. [42] also identified the empowerment of nurses through training, nurses’ performance monitoring, and teamwork promotion as key strategies that NM can implement to prevent MNC. Additionally, a systematic review conducted by He et al. [63] emphasized the importance of promoting communication skills within nursing teams and between nurses and hospitalized persons to reduce MNC. A case study conducted by Avallin et al. [64] also concluded that active patient involvement and effective nurse–patient communication can reduce the occurrence of MNC. 

Most of the knowledge about MNC is derived from the MISSCARE Survey developed in the United States of America (USA), which relies on nurses’ self-reports that may be susceptible to response bias and social desirability [65], while evidence resulting from hospitalized persons’ perceptions remains limited. Vincelette et al. [66] warned that the contributions of hospitalized persons and their family members are rarely considered in studies aiming to identify nursing care activities that are “missed” during nurses’ shifts. Some authors affirm the ability of hospitalized persons to recognize safety-related issues that healthcare professionals may overlook during the provision of care [67]. During hospitalization, persons are generally attentive to healthcare professionals and can identify safety issues, such as difficulties related to entry into and discharge from the healthcare system, systemic problems that are cumulative and omissive, especially failures to address people’s concerns [68]. In addition, hospitalized persons may become actively involved in Patient Safety initiatives motivated by several factors: (1) optimizing safety, costs, and care processes, (2) improving relationships between hospitalized persons and healthcare professionals, and (3) improving their own care experiences and outcomes [69]. The experience of care reported by hospitalized persons has long been considered an essential quality indicator and is associated with the provision of nursing care [70]. 

To assess hospitalized persons’ perceptions of MNC, Kalisch and Williams developed the MISSCARE Survey-Patient [71], a medium-range theory relevant for analyzing the fundamental characteristics that contribute to the occurrence of MNC reported by nurses [46]. This theory considers that structural antecedents may positively or negatively influence the nursing process and nurses’ internal processes, including team norms, decision-making processes, internal values and beliefs, and professional habits [46]. Based on this framework, MNC may occur not only during the implementation phase of nursing interventions but also in other phases of the nursing process [72]. Nurses’ decision-making processes are influenced by the availability of human resources, the number, training, and experience of nursing staff, the availability of support for the team, and material resources and operational equipment [73]. When these factors are compromised, nurses reassess and prioritize nursing care based on human resources [73]. Hospitalized persons and their families may also influence nurses’ decision-making through requests or demands that affect the prioritization of care activities [72]. 

Hospitalized persons and their families are the only elements that remain constant throughout the care process and can therefore play a critical role in maintaining the continuity of care [74], particularly when they are involved with and empowered by nurses [75].Therefore, hospitalized persons should be involved in discussions regarding nursing care needs and prioritization processes, especially when nurses face difficulties in responding to all hospitalized persons’ needs [76]. We conceptualize hospitalized persons as partners in the provision of care and experts in their own health conditions [77]. This point of view is consistent with the WHO’s perspective, which recognizes hospitalized persons and their families as Co-Producers of Health within healthcare systems [78]. Consequently, in this study, the designation of hospitalized persons is adopted following the recommendation by the Institute of Medicine [79], replacing the use of the term *patient* in order to promote a more comprehensive perspective and a broader understanding of outcomes and impacts relevant to the population.

### 2.1. Study Rationale

Within the taxonomy of nursing errors, MNC can be classified as a phenomenon characterized by failures in prevention resulting from inadequate monitoring or follow-up [80]. Some authors argue that NMs should be informed about the occurrence of MNC in the units that they manage [81]. For this purpose, NMs need to develop a non-punitive environment that facilitates the reporting of MNC situations [82]. Cordeiro et al. [83] argue that the best method for assessing MNC is direct observation. However, this approach is costly and too expensive. The involvement of hospitalized persons is one of the strategies recommended by Kalisch et al. [84] to assist nurses in assuring the completion of all aspects of nursing care. Conceptually, patient involvement consists of a partnership between hospitalized persons and healthcare professionals across multiple levels of the healthcare system, including direct care, service design, organizational governance, and health policy development [85]. Consequently, the availability of an assessment tool capable of capturing the degree of MNC experienced by hospitalized persons represents an accessible source of information that may support NMs to design interventions to mitigate the occurrence of MNC. 

In Portugal, an adapted version of the MISSCARE Survey assessing nurses’ perspectives was found [86]. However, to date, no translated and cross-culturally adapted version of the MISSCARE Survey-Patient [71] has been identified. Based on this gap, the following research question was formulated:

- **Does the translation and cross-cultural adaptation of the MISSCARE Survey-Patient for the Portuguese population demonstrate adequate measurement properties?**

Adequate measurement properties are a prerequisite for assessment instruments to be useful and interpretable [87].

### 2.2. Aim and Objective of the Study

The aim of this study was to obtain a valid and reliable version of the MISSCARE Survey-Patient [71] for assessing the degree of MNC in Portuguese hospitals. To achieve this aim, the following objective was defined: to translate, adapt, and cross-culturally validate the MISSCARE Survey-Patient for the Portuguese population and to analyze the quality of its measurement properties assessed within the scope of this study. Measurement properties of an assessment instrument are defined as the set of quantitative and qualitative evidence produced by researchers during the development of assessment instruments or during their translation, adaptation, and validation for use in a different cultural context [88,89,90]. 

This study represents the first Patient-Reported Experience Measure (PREM) specifically focused on nursing care available in Portugal. The instrument will provide Portuguese NMs with information on the experience of hospitalized persons regarding nursing care, enabling them to improve the quality and safety of nursing practice environments. 

The cross-cultural adaptation and validation of this measurement instrument provide insights into the MNC experienced by hospitalized persons in Portugal and, consequently, supports the development of complex interventions in Nursing Management to improve the safety of nursing care across several contexts of the Portuguese healthcare system. 

The objective of this study is aligned with Sustainable Development Goal (SDG) 3, which focuses on ensuring access to safe and quality essential health services for all [91].

## 3. Materials and Methods

### 3.1. Study Design

This is a validation study with a quantitative methodology and a non-interventional, descriptive, cross-sectional research design [92]. 

This study was developed based on the guidelines proposed by Cruchinho et al. [93] and is part of a broader research project on the effectiveness of a complex intervention for improving Patient Safety through the promotion of patient participation in Nursing Handover. The COSMIN (COnsensus-based Standards for the selection of health status Measurement INstruments) reporting guideline for studies on measurement properties of patient-reported outcome measures: version 2.0 [94], (Table S1) and the STROBE statement, refs. [95,96] were used to guide the reporting of this study (Table S2). COSMIN is a consensus-based guideline to evaluate the methodological quality of studies on the measurement properties of health status measurement instruments based on an international Delphi study in 2019–2021 [94]. Although aimed at Patient-Reported Outcomes Measures (PROMs), it constitutes a benchmark for assessing the methodological quality and measurement properties of Patient-Reported Experience Measures (PREMs) [97]. PREMs measure hospitalized persons’ personal experience of the healthcare that they receive [98,99] and differ from satisfaction questionnaires in that they focus on objective experiences, rather than subjective opinions [100]. Mastery of the methodological approaches established by COSMIN is a fundamental prerequisite for validation studies involving PROMs and PREMs [101].

### 3.2. Ethical Issues

Permission was obtained to translate and cross-culturally validate the MISSCARE Survey-Patient [71] from the authors and from the publisher Wolters Kluwer, which holds the intellectual property rights to the American Journal of Medical Quality, where the original instrument-development study was published. 

This study was approved by the Ethics Committee for Health of Hospital de Cascais Dr José de Almeida (approval number EC09-2025 20/CE 11/06/2025) and was conducted in accordance with the Declaration of Helsinki [102]. Before consenting to participate, all participants were informed of the study objectives, methods, research-data-retention period, absence of risks, and that participation would provide individuals admitted to Portuguese hospitals with a measurement instrument that would enable hospital NMs to ascertain the degree of nursing care omission and any adverse events experienced during their hospitalization. 

All participants provided written and voluntary informed consent. No personal identifiers were collected, and all data were anonymized to ensure participants’ privacy and confidentiality. Throughout the study, all ethical principles were strictly followed [103,104].

### 3.3. Survey Description

The MISSCARE Survey-Patient [71] is an instrument derived from the original MISSCARE Survey, which was developed to assess MNC and its underlying reasons for its occurrence from nurses’ perspectives. The MISSCARE Survey-Patient [71] represents a second-generation development of the original instrument designed for specific contexts and targets [105], enabling the assessment of hospitalized persons’ perceptions regarding the occurrence of MNC and the care environment reported by nurses, namely whether nursing care was provided during their hospitalization [76]. The instrument comprises three sections: (1) nursing care activities; (2) adverse events sensitive to nursing care experienced by hospitalized persons; and (3) sociodemographic data. 

The nursing care activities section includes 13 items with a five-point response scale to assess two dimensions: Basic Care and Communication (from 1 = never to 5 = always), and Time to Response (from 1 =< 5 min to 5 => 30 min) [71]. The Communication dimension represents the frequency of communication received from the nursing team, while Basic Care refers to the volume of basic nursing care received [106]. Regarding the Time to Response dimension, all items were reverse-coded so that lower scores represent higher levels of nursing care omission. 

The sociodemographic section includes the following variables: (1) number of days of hospitalization; (2) whether there were previous hospitalizations; (3) number of hospitalizations in the last five years; (4) age; (5) gender; (6) highest education level; (7) hospital unit of admission; and (8) whether the person is accompanied by a significant other. 

The total score of the instrument is obtained by summing the items, resulting in values ranging from 13 to 65. Higher scores indicate lower levels of perceived MNC among hospitalized persons. 

The final section asks participants whether they experienced any of the following nurse-sensitive adverse events during their hospitalization: (a) falls; (b) pressure sores/ulcers; (c) medication errors; (d) new infections; (e) empty IVs; and (f) IV leakage. In addition, it included a question addressed to “other problems”, allowing participants to report other adverse events that had occurred [71]. In addition, a question assessing satisfaction with nursing care was included to evaluate Convergent Validity. 

To date, the MISSCARE Survey-Patient [71] has only been translated and cross-culturally adapted into Danish [9], Chinese [107], and Turkish [108]. Authors of the original instrument reported the evaluation of some measurement properties of the instrument. One of these properties was Content Validity, assessed through a focus group with nurses and hospitalized persons from medical-surgical units based on the clarity and relevance of the items. For nurses and hospitalized persons, the authors reported CVI of 0.890 and 0.880, respectively. The authors also analyzed the Convergent Validity of the instrument by comparing the results of the MISSCARE Survey-Patient [71] with a question about satisfaction with nursing care. They also reported that high levels of overall satisfaction with nursing care were correlated with low levels of MCN (r = −0.25; *p* < 0.001). Convergent Validity was recognized by Palese et al. [105] as one of the least evaluated properties by the different instruments that have been developed and validated to measure MNC. In previous validation studies of the MISSCARE Survey-Patient [71], the question about satisfaction with nursing care during hospitalization was included in the demographic characteristics section, along with questions about adverse events sensitive to nursing care [9].

In the authors’ previous study [71], Construct Validity was assessed through exploratory factor analysis followed by confirmatory factor analysis. The exploratory factor analysis obtained a three-factor solution explaining 59.2% of the variance in perceptions of MNC among hospitalized persons: (1) Communication (with five items); (2) Response Time (with four items); and (3) Basic Care (with four items). Confirmatory factor analysis supported the adequacy of this model. Test-Retest Reliability was analyzed in a random sample of 30 hospitalized persons who completed the questionnaire during hospitalization and again two weeks after discharge. This analysis yielded an overall test-retest coefficient of 0.818. Finally, Internal Consistency was assessed using Cronbach’s α, with a value of 0.838 for the overall instrument and values ranging from 0.708 to 0.834 for the three subscales [71]. 

### 3.4. Study Organization

**Figure 1:**
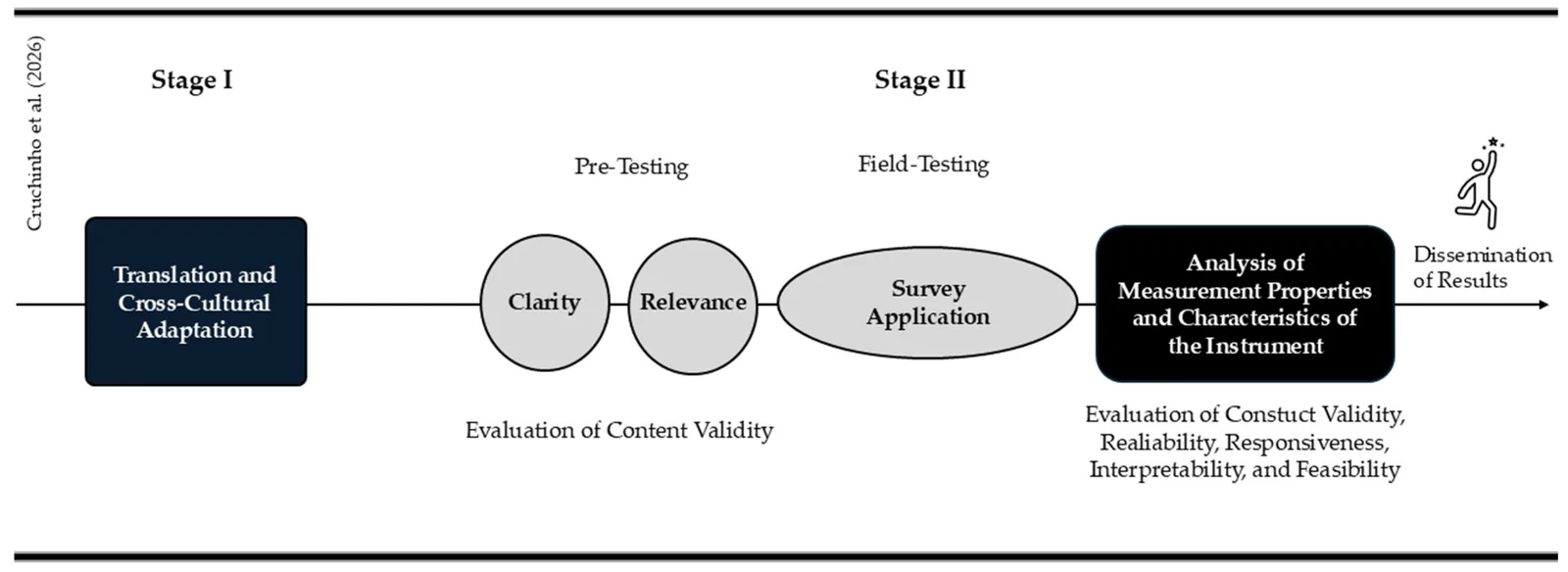
Overview of the study.

This study was conducted between July 2025 and February 2026 in two stages: (1) translation and cross-cultural adaptation of the MISSCARE Survey-Patient [71] and (2) field testing and psychometric validation of the pre-final Portuguese version (MISSCARE Survey-Patient-PT). Figure 1 presents an overview of the study.

#### 3.4.1. Stage I: Translation and Cross-Cultural Adaptation of MISSCARE Survey-Patient [71]

This phase of the study was conducted prior to fieldwork at the host institution. It included the translation of the instrument from the source language (English) into the target language (Portuguese). Two bilingual translators whose native language was Portuguese collaborated in this procedure; one of them was familiar with the construct of the instrument and the other unfamiliar with health terminology. Each translator independently produced a translated version of the MISSCARE Survey-Patient [71]. The research team then analyzed the ambiguities and discrepancies identified and synthesized the two translations into a single version. The synthesized version was then translated from the target language (Portuguese) into the source language (English), independently and by two bilingual translators whose native language was English. The research team then compared the back-translated version with the original version of the instrument, the synthesized version, and the two initial translation versions to identify any ambiguities and discrepancies.

This process resulted in a translation proposal for each item in the instrument. At this stage, discrepancies were found in the translations of “IV running dry” and “IV leaking into your skin”. An attempt was made to contact one of the authors who developed the original instrument to clarify the intended meaning of these expressions, but no response was obtained. Based on the literature review, “IV running dry” corresponds to a predictable occlusion of the intravenous system [109], which may result in air embolism, a complication due to the inadequate control of serum flow [109,110]. Vinan-Vega et al. [111], based on a case report of a 47-year-old woman, warned that this complication may be more common than expected. Furthermore, some authors recommend preventing air entry into the intravenous system by avoiding situations where the IV bag runs dry and by replacing infusion solutions in a timely manner [112]. Based on this evidence, the research team proposed translating this item as “During this hospitalization, did you happen to have any empty IV bags and/or medication packaging that were not removed?”. Additionally, the literature indicates that “IV leaking into your skin” is defined as the unintentional infusion of non-vesicant solution into the surrounding tissues [113]. When the infusion causes blisters, ulceration, and sloughing, it is called extravasation and is associated with the presence of a vesicant solution outside the vein [114]. To detect infiltration early, before it becomes severe, nurses need to monitor the IV catheter-insertion site [115]. Consequently, the following wording was proposed for the item: “During this hospitalization, did the IV drip leak out of your vein and into your skin?”. 

This translation proposal was then presented, discussed, and agreed upon by a Multidisciplinary Committee formed on the basis of a joint analysis of the original version, the two translation versions, the summarized version, and the two back-translation versions. This Multidisciplinary Committee operated through an online meeting and included the four translators who participated in the translation procedures of the MISSCARE Survey-Patient [71], two members of the research team, one monolingual, and four nurses from the host institution, with two of them having nursing coordination roles. To achieve consensus within the Multiprofessional Committee, the dual-focus technique was applied, involving an iterative process in which the wording of the instrument items was reviewed and discussed in both languages by individuals with expertise in the construct being measured [116]. Within the Multiprofessional Committee, this perspective was provided by monolingual nurses and representatives of the target population. The purpose of the dual-focus approach was to ensure agreement among all translators regarding the semantic and conceptual equivalence of the instrument [116]. Members familiar with the Nursing Missed Care construct contributed by examining the potential meanings of terms and proposing wording that more accurately reflected the intended concepts. During the Multiprofessional Committee meeting, all identified discrepancies and divergent interpretations across the items were systematically discussed. A member of the research team facilitated the discussions, encouraging the translators to reach consensus on the wording that best preserved semantic equivalence for every item. Once agreement had been achieved among the translators, members familiar with the construct were invited to comment on the proposed wording and suggest alternative expressions that better represented the Nursing Missed Care construct. Table S3 presents the different versions of the instrument produced during each stage of the translation and cross-cultural adaptation process.

#### 3.4.2. Stage II: Field Testing and Psychometric Validation of the Final Version of the MISSCARE Survey-Patient-PT

This phase included two pre-tests of the adapted version in the Multidisciplinary Committee, followed by field testing of the MISSCARE Survey-Patient-PT in the medical and surgical departments of the hospital where the study was conducted. The first pre-test was aimed at assessing the clarity of the items and involved a sample of hospitalized persons as experts, while the second pre-test consisted of assessing the cultural relevance of the items, which included nurses providing direct patient care. Both pre-tests were conducted using a paper questionnaire, followed by individual debriefing. These questionnaires contained two sections, one with the items of the assessment instrument and the other with sociodemographic data. In the clarity assessment questionnaire, the following statement was replicated alongside each item of the instrument: “In my opinion, the previous question is” (mark with an X), with two possible answers: “(a) clear” and “(b) unclear” followed by the statement “I consider it ‘unclear’ because” preceded by a text box for participants to write down the least clear elements they found. 

The relevance assessment questionnaire also included two similar statements for each item: “In my opinion, the previous question is” (mark with an X), with four possible answers, “(a) not relevant; (b) slightly relevant; (c) quite relevant; and (d) highly relevant”. The second question was also preceded by a free text box for participants to fill in: “I consider it to be ‘not relevant’ or ‘not very relevant’ because”. The instruction for the clarity assessment questionnaire was as follows: “Carefully read each of the sentences below, marking whether or not they are clear”. In the relevance assessment questionnaire, we provided the following instruction: “Carefully read each of the questions, marking whether, in your opinion, they are relevant or not to the provision of nursing care in Portugal. Relevance was understood as the degree to which a particular nursing behavior or problem might be encountered in the provision of care by Portuguese nurses”. The pre-test relevance questionnaire was given to nurses at a time during their shift when they had a lighter workload.

For the clarity pre-testing, hospitalized persons were recruited according to the following criteria: (1) hospitalization for at least five days; (2) age ≥ 18 or over; (3) agreed to participate voluntarily; (4) ability to communicate orally and in writing [117]; (5) fluency in Portuguese; and (6) absence of neurovegetative diseases. Criteria related to academic qualifications were not defined to ensure that the questionnaire was clear to all hospitalized individuals regardless of their level of education. 

In turn, for the relevance pre-testing, the criteria defined were as follows: (1) be a nurse in one of the medical or surgical departments of the institution hosting the study; (2) agree to participate voluntarily in the study when invited by the researcher; (3) have at least two years of professional experience in the department where they work. This criterion was defined because, according to Benner et al. [118], nurses with two or more years of professional experience have already acquired considerable experience and clinical knowledge in nursing practice and since the assessment of the cultural relevance of the items in the measurement instrument requires the judgment of a nurse who is no longer a novice in general nursing practice. Because the MNC is influenced by nurses’ decision-making processes [46], only nurses were included.

Regarding the sample size, for both the clarity pretest and the relevance pretest, we followed the guidelines of Wild et al. [119] and Ortiz-Gutiérres and Cruz-Avelar [120], who recommend a minimum of eight participants for individual debriefing. In both pre-tests, we recruited participants based on the convenience sampling method, five from medical services and another five from surgical services. Potential participants were identified with the collaboration of nurse coordinators from the participating departments. To improve and refine the questionnaire items, a face-to-face individual debriefing was conducted to explore why participants chose the option “not clear” in the case of the clarity pre-test and the options “not relevant” and “not very relevant” in the relevance pre-test [121]. In this individual debriefing, the principal investigator used a reparative approach with verbal reactivities and retrospective probing to identify potential problems in the wording of the items that could lead to response errors [122]. The principal investigator asked the following question in response to the answers “not clear,” “not relevant,” and “not very relevant”: “Can you explain what you mean by this answer?” The researcher was particularly attentive to the following: (1) terms that were complex to understand; (2) inappropriate assumptions about the respondent or the context of the situation represented in the question. The participants’ responses were recorded in field notes.

**Figure 2:**
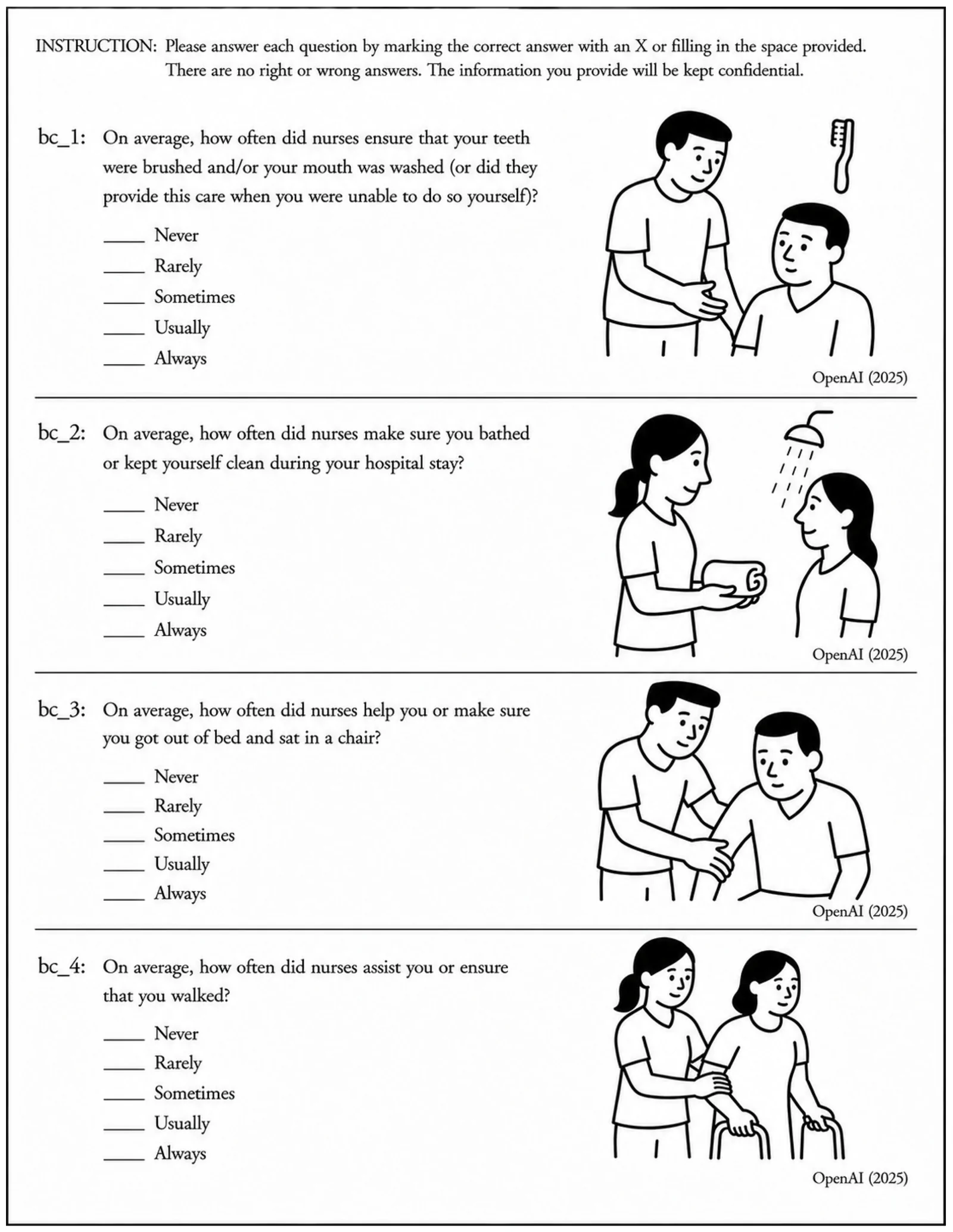
Example of items that measure MNC in relation to Basic Care.

To assess the Face Validity of the MISSCARE Survey-Patient [71], the following question was included in the pre-test relevance questionnaire: “Do you think any of the items in the questionnaire may be unclear and difficult for hospitalized persons to answer?”. Although Lim [123] recommends that Face Validity should be assessed after Content Validity and before field testing, in this study, this question was included in the pre-test relevance assessment to optimize data-collection time. To facilitate an understanding among hospitalized persons with low or limited health literacy, the researchers included an illustrative image for each item in the measurement instrument. Figure 2 presents examples of items that measure MNC and are related to basic nursing care.

For the field testing of the final version of the MISSCARE Survey-Patient-PT, the following inclusion criteria were established for hospitalized persons: (1) have been hospitalized for at least five days; (2) are aged 18 years or older; (3) agree to participate voluntarily in the study when invited by the researcher; (4) have the ability to communicate, namely in receiving and producing oral and written messages [117]; (5) are fluent in Portuguese; and (6) do not have a diagnosis of neurovegetative diseases. The sample size to be achieved was guided by the criterion of 10 participants per survey item [124]. People who did not meet these criteria were excluded from the study. 

A consecutive sampling strategy [125] was adopted. During each data-collection visit, the researcher responsible for data collection, in collaboration with the coordinating nurses of the participating wards, prospectively screened hospitalized patients for eligibility according to the predefined inclusion and exclusion criteria. The identification of potential participants who met these criteria was carried out in collaboration with the coordinating nurses of the services involved. Data collection was performed by distributing a paper questionnaire together with detailed information on how to complete it, after the informed consent form had been signed. The researcher responsible for data collection was available to answer questions and provide guidance as needed during the data-collection period. In addition, sufficient time was provided for participants to complete the questionnaire at their convenience. At the time of questionnaire collection, the researcher verified the completion of the MISSCARE Survey-Patient-PT items, inviting participants to complete any items that were left blank or unclear, where applicable.

### 3.5. Study Settings

This study was conducted in the medical and surgical units of a medium-sized general hospital [126,127], in the Lisbon metropolitan area (Portugal). This hospital includes the Chief Nurse Executive as a formal member of the organization’s highest decision-making body, and the nursing services have a horizontal organizational structure led by nurse coordinators. From a financial standpoint, this hospital is a public–private partnership (PPP), characterized by collaboration between the public and private sectors [128] to increase not only the availability of resources but also the diversity of knowledge and make the innovation process less costly and expensive, more efficient, effective, shorter, and with greater reach [129]. The medical service comprises three wards and the surgical service two, each with a capacity of 27 beds. In both services, nurses work eight-hour shifts, with a written handover system. Both services have an organizational culture geared toward continuous improvement resulting from accreditation by Joint Commission International (JCI), which includes a set of International Patient Safety Goals aimed at certain indicators sensitive to nursing care and improving equity in health care [130]. To describe the research context, the domains of Piña et al.’s Framework to Describe Health Care Delivery Organizations and Systems were followed [131]. The research context of this study is described because research results are often moderated by the context in which the research is conducted [132].

### 3.6. Data Analysis

#### 3.6.1. Descriptive Statistics

The characteristics of the samples involved in the pre-tests of the pre-final version of the MISSCARE Survey-Patient-PT are described using absolute numbers. Regarding the field testing of the final version, the characteristics of the study sample are presented as absolute frequencies (n) and percentages (%) for categorical variables and as means and standard deviations (SDs) for continuous variables. Descriptive statistics of the MISSCARE Survey-Patient-PT items were used in this study to characterize the study sample.

#### 3.6.2. Analysis of Psychometric Properties and Characteristics of the Instrument

Measurement or psychometric properties are characteristics of assessment instruments that allow the validity and reliability of the results obtained in a specific context to be evaluated [133]. They correspond to a set of quantitative and qualitative evidence produced during the development of assessment instruments and subsequently during their translation, adaptation, and cross-cultural validation [89,90,134], providing information on their quality within specific populations and contexts [87]. Based on the COSMIN methodology, four domains of psychometric properties were defined, comprising a variable number of psychometric properties: (1) Content Validity; (2) Construct Validity; (3) Reliability; and (4) Responsiveness [135]. In addition, based on the same guideline, two characteristics of the instrument were evaluated: (1) Interpretability and (2) Feasibility. 

Content Validity. To support decisions regarding potential improvements in clarity and the partial or complete replacement of items in the MISSCARE Survey-Patient-PT, the Content Validity Index (CVI) was calculated and the justifications and suggestions for wording obtained during individual debriefing were analyzed. The CVI calculation is a method of assessing inter-rater agreement, which is used by researchers to quantify Content Validity [136]. Two approaches were used to determine the CVI: (1) calculation of the CVI for each item (I-CVI) in the pre-final version of the MISSCARE Survey-Patient-PT and the total assessment instrument (S-CVI/Ave) and (2) the Universal Agreement Index (UA and S-CVI/UA). To determine the S-CVI/Ave, the following formula was used: S-CVI/Ave = (sum of I-CVI scores)/(total number of items), while the S-CVI/UA was determined based on the quotient of the sum of the UA values by the total number of items [137]. 

UA is a method of scoring evaluator agreement by assigning a (1) when an item obtains 100% agreement and (0) when agreement is less than 100% [137]. To determine the CVI for each item in the relevance pretest, the response options “not relevant” and “somewhat relevant” were recoded as “not relevant,” and the options “quite relevant” and “highly relevant” were recoded as “relevant.” In this study, minimum reference values of 0.80 were adopted for the I-CVI [136] and S-CVI/UA [138] and 0.90 was adopted for S-CVI/Ave [136]. To facilitate the assessment of clarity and relevance regarding adverse events sensitive to nursing practice, the initial formulation was broken down into independent questions [137]. In the original instrument, these questions had a common formulation. 

Face Validity. After analyzing Content Validity, the MISSCARE Survey-Patient-PT was analyzed for Face Validity. Face Validity refers to evidence that the items of an instrument are perceived as clear, understandable, relevant, and easy to answer and are non-judgmental, non-intrusive, or non-distressing to the target population [139]. The Item-Level Face Validity Index (I-FVI) and Scale-Level Face Validity Index (S-FVI/Ave) were computed. To interpret these indices, we used minimum limits of 0.80 for I-CVI and 0.90 for S-CVI/Ave [136].

Construct Validity. This comprised the assessment of Structural Validity through Exploratory Factor Analysis (EFA) and Confirmatory Factor Analysis (CFA), with both analyses initially conducted on the same dataset, consistent with the approach proposed by Van Prooijen and Van Der Kloot [140]. Structural Validity can also be referred to as Factor Validity and is defined as a form of content-related validity that can be assessed based on the number of factors, structures, or dimensions that underlie a set of items in an assessment instrument [141]. It refers to the scoring algorithm of the assessment instrument [142]. The Kaiser–Meyer–Olkin (KMO) test and Bartlett’s sphericity test were used to assess the suitability of the variables for factor analysis. KMO values ≥ 0.70 and the significance of the Sphericity Tests (*p* < 0.001) are indicators of an adequate sample size and support the performance of EFA and CFA [143,144]. The decision on the number of factors to retain was based on the criterion of an eigenvalue > 1 [145]. The varimax method was used to extract factors in order to maximize the variance within the factors [145]. Four decision criteria were used in EFA: (1) the saturation coefficient or factor loading with a cut-off point > 0.40 [145,146]; (2) selecting the factor with the highest factorial weight; (3) excluding items with communalities < 0.30; and (4) the percentage of variance of the explanatory model between 50% and 60% for the Social Sciences [146]. This reference interval is ontologically congruent with the researchers’ assumption that nursing is a social practice, as it is contextualized not only in the individual situations of hospitalized persons and nurses, but above all in the continuous structure of practical situations, in which nurses influence and are influenced by each other’s practice [147]. IBM^®^ SPSS^®^ Statistics software, version 29.0 (IBM Corp., Armonk, NY, USA), was used to obtain the factor structure [148].

Subsequently, a CFA was performed on the factor structure obtained in the EFA, utilizing the maximum-likelihood approach and various commonly used goodness-of-fit measures. To provide adequate evidence of the adjustment model, it is necessary to report at least three indices [149]. According to Preacher [150], these indices should comprise an absolute index, a parsimonious index, and an incremental index. Consequently, in this study, the overall quality of the factorial model adjustment was evaluated based on the following reference values for the following indices: (1) Chi-square test (X^2^)/df ≤ 3.00 [151]; (2) Parsimony Comparative Fit Index (PCFI) ≥ 0.50 [152], and (3) the Comparative Fit Index (CFI) with higher values indicating better fit [149]. The model adjustment was produced by the software IBM^®^ SPSS^®^ Amos^TM^, Version 29.0 (IBM Corp., Armonk, NY, USA) [153], based on theoretical considerations [154].

Although the COSMIN guideline [94] includes Differential Item Functioning (DIF) and Hypothesis Testing in the assessment of Construct Validity in its assessment of Cross-Cultural Validity, these analyses were not planned in this study because of the following: (1) the sample consists only of participants from the Portuguese population and (2) no PREM focused on constructs similar to the MNC [155] is yet available in Portugal. The latter reason also led the researchers not to plan the assessment of the Concurrent Validity of the assessment instrument.

Reliability. This measurement property is defined as the degree to which hospitalized persons’ scores are the same for the same measurements under various conditions: (a) using different sets of items from the same measurement instrument (Internal Consistency); (b) over time (Test–Retest); (c) by different investigators on the same occasion (Inter-Rater) or by the same investigators on different occasions (Intra-Rater) [142]. In this study, we evaluated this measurement property based on Internal Consistency and the Measurement Error. Internal Consistency Reliability is defined as the degree of the correlation, association, or covariance of a set of items developed to measure the same construct or concept [156]. To assess Internal Consistency, we used the following: (1) Cronbach’s α; (2) Mean Inter-item Correlation; and (3) Amplitude of the Item-total Correlations (ITC).

Cronbach’s α provides information on the degree to which items produce similar scores [157]. Cronbach’s α was calculated in total and for each subscale separately. The interpretation based on Cronbach’s α value was based on the following scale: (a) α < 0.60 (weak value); (b) 0.60 ≤ α < 0.70 (questionable value); (c) 0.70 ≤ α < 0.80 (acceptable value); (d) 0.80 ≤ α < 0.90 (very good value); and (e) ≥0.90 (excellent value) [158]. For cross-culturally adapted instruments, a Reliability of 0.70 is considered acceptable [159]. 

The Mean Inter-Item Correlation (MIIC) assesses the degree to which items on a scale are related to items on the same scale and assess the same construct [160]. In the MIIC assessment of the subscales or dimensions of the MISSCARE Survey-Patient-PT, the following reference values were used: (1) values < 0.30 suggest little congruence with the instrument’s construct; (2) values ≥ 0.30 and ≤0.70 suggest congruence with the construct; and (3) values > 0.30 suggest redundancy with the construct [161]. MIIC values for the total scale between 0.20 and 0.40 mean that the items are reasonably homogeneous and contain enough unique variance to not be considered isomorphic with each other [160]. For instruments that assess broad higher-order constructs, MIIC values between 0.15 and 0.20 are considered acceptable [162].

ITC is defined as the correlation between an item on an assessment instrument and the total score for that instrument [163] and provides information on the degree of homogeneity of the items that make up a scale [164]. In the ITC assessment, values > 0.20 were considered acceptable [165].

The Reliability of a measurement can be affected by specific factors that affect participants [163]. Measurement Error refers to the potential bias and variation generated by these factors, namely: (1) the researcher collecting the data; (2) the instrument used to collect the data; (3) the participants being evaluated and the mode of data collection (whether in-person or self-administered) [166]. To evaluate Measurement Error, we calculated the Standard Error of Measurement (SEM). SEM is an estimator that quantifies Measurement Error related to Reliability [167]. It is defined as the standard deviation of Measurement Errors associated with the scores of an assessment instrument for a given group of respondents [168]. It is used to report individual scores and score differences in assessment instruments [169]. SEM was calculated using the formula $SD\sqrt{1-r}$ (*SD* = Standard Deviation; *r* = instrument’s reliability) [167]. The more reliable a measuring instrument is, the smaller the SEM will be, and vice versa [170]. Low SEM values for the entire instrument or its dimensions, good Measurement Error characteristics [171].

Convergent Validity. Convergent Validity assesses the degree to which two theoretically related measures are associated [123]. It is a strategy that reinforces Construct Validity [172]. In this study, Convergent Validity was examined by correlating the scores of the MISSCARE Survey-Patient with a question assessing satisfaction with nursing care included in the questionnaire. Convergent Validity is generally considered acceptable when correlations between theoretically related measures exceed 0.50 [173].

Responsiveness. This is defined as the sensitivity of a given assessment instrument to detect changes over time or after an intervention [174]. This is an important characteristic because assessment instruments may have acceptable reliability and validity but not be sensitive (or responsive) [175]. The Responsiveness of the MISSCARE Survey-Patient-PT was assessed based on the Minimal Important Change (MIC). This is the smallest change in a score within the construct being measured that hospitalized persons perceives as important [176]. The Responsiveness of a measurement instrument was evaluated by comparing the MIC and the Smallest Detectable Change (SDC). This is the smallest change that can be detected by the measuring instrument, apart from the Measurement Error [176]. If SDC < MIC, the instrument’s responsiveness is confirmed [177]. SDC was calculated based on the formula *SDC* = *SEM* × 1.96 × $\sqrt{2}$ [178]. The MIC was estimated using a distribution-based approach [179] and calculated based on the formula MIC = SD × 0.5 [177].

The quality of measurement properties was interpreted according to the reference values described above. 

Interpretability. According to COSMIN guidelines, Interpretability is considered a characteristic of assessment instruments rather than a measurement property [87]. It consists of the degree to which qualitative meaning can be attributed to the scores of an assessment instrument or to changes in scores [180]. To assess Interpretability of the MISSCARE Survey-Patient-PT, potential floor and ceiling effects were evaluated for the total score and for each of the three dimensions. The ceiling effect was considered present when more than 15% of hospitalized persons obtained the maximum score, and a floor effect was considered when more than 15% of hospitalized persons obtained the minimum score [181]. Ceiling and floor effects may compromise Reliability, as they limit the ability of the instrument to discriminate between participants with very low or very high scores [119].

Feasibility. It is defined as the ease of applying a PROM or PREM in the intended context, considering constraints such as time and cost [182]. Feasibility depends not only on the instrument, but also on the age and functional abilities of hospitalized persons [183]. As with Interpretability, Feasibility is considered a practical aspect of assessment instruments rather than a measurement property [182].

## 4. Results

First, the results of the Content Validity assessment carried out during the clarity and relevance pre-tests are presented. Next, the remaining psychometric properties and instrument characteristics obtained during field testing of the final version are reported.

### 4.1. Pre-Testing of Clarity and Relevance of the Pre-Final MISSCARE Survey-Patient-PT

#### 4.1.1. Characteristics of Pre-Test Participants

Ten hospitalized persons participated in the clarity pre-test (five female and five male). Most participants were aged over 65 years, had a high school education or higher, and had been hospitalized two to five times in the previous five years. 

In turn, 10 nurses participated in the relevance pre-test. Most were women, aged between 25 and 34 years old, with six or more years of professional experience; three had postgraduate nursing education.

#### 4.1.2. Content Validity 

An S-CVI/Med value of 0.98 and an S-CVI/UA value of 0.84 were obtained for the overall instrument, showing that hospitalized persons understood the PREM instructions, items, and response options as intended. Only two items revealed disagreement among participants, despite an I-CVI of 0.90: (1) item co_2: “On average, how often did nurses discuss your treatment with you?” (Em média, com que frequência os enfermeiros discutiram consigo o seu tratamento?) and (2) item co_3: “On average, how often did nurses give you information about tests (e.g., X-rays, MRI, CT scan) and/or procedures you underwent during your hospitalization (namely, when they would be performed and what they consisted of, etc.)?” [Em média, com que frequência os enfermeiros lhe deram informações sobre os exames (por exemplo, raio-X, ressonância magnética, TAC) e/ou procedimentos que realizou durante o internamento (nomeadamente, o momento em que seriam feitos e no que consistiam, etc.)?]. 

During individual debriefing, item co_2 was marked as “unclear” because it was the doctors who discussed the treatment with the hospitalized persons, rather than the nurses. Similarly, item co_3 was marked as “unclear” because it was the doctors who provided information about the diagnostic results of the tests. These observations were discussed with two collaborating NMs at the host institution. The research team then refined the wording of these items to improve comprehensibility: co_2: “On average, how often did nurses discuss your care plan with you?” (Em média, com que frequência os enfermeiros discutiram consigo o seu plano de cuidados?) and co_3: “On average, how often did nurses provide you with information about the tests and/or procedures you underwent during your hospitalization (namely, when they would be performed and what they consisted of, etc.)?” [Em média, com que frequência os enfermeiros lhe forneceram informações sobre os exames e/ou procedimentos que realizou durante o seu internamento (ou seja, quando seriam realizados e em que consistiam, etc.)?].

During individual debriefing, some participants also suggested improvements to two items in the section addressing the experience of adverse events during hospitalization: (1) “During this hospitalization, did you happen to have any empty IV bags and/or medication packaging that were not removed?” (Neste internamento aconteceu-lhe ficar com embalagem(s) de soro e/ou medicação vazia(s) sem ser removida(s)?), and (2) “During this hospitalization, did the IV drip leak out of your vein and into your skin?” (Neste internamento aconteceu-lhe ficar com o soro a sair fora da veia e ir para a pele?). These two items were also discussed with the nurses collaborating in the study, leading the research team to improve the wording of these items to “During this hospitalization, did your IV drip stop because it was completely empty?” (Neste internamento aconteceu-lhe ficar com o soro sem pingar por estar completamente vazio?) and “During this hospitalization, did your hand or arm become swollen because of the IV?” (Neste internamento aconteceu-lhe ficar com a mão ou braço inchados por causa do soro?). Based on these modifications, it was considered that the PREM was adequately worded. Table A1 shows the content validity indices for clarity obtained for each item and for the pre-final version of the MISSCARE Survey-Patient-PT.

For the overall instrument, S-CVI/Med and S-CVI/UA indices of 1.00 were obtained, indicating that all items of the instrument were considered relevant in PREM. Table A2 presents the content validity indices obtained for each item and for the pre-final version of the MISSCARE Survey-Patient-PT, in relation to relevance.

#### 4.1.3. Face Validity 

The MISSCARE Survey-Patient-PT showed strong Face Validity, achieving an excellent average Scale-level Face Validity Index (S-FVI/Ave) of 1.00, surpassing the recommended threshold of 0.90 [136]. Based on participants’ responses, each item was individually evaluated with an Item-level Face Validity Index (I-FVI) of 1.00, clearly meeting or exceeding the accepted standard of 0.80 [136]. Only two participants identified the items “Did you suffer any skin lesions or pressure ulcers during this hospitalization?” (Neste internamento aconteceu-lhe alguma lesão na pele ou úlcera por pressão?) and “Did you suffer any skin lesions or pressure ulcers during this hospitalization?” (Neste internamento aconteceu-lhe alguma infeção adquirida durante a permanência no hospital?) as potentially difficult for hospitalized persons. These participants explained in the individual debriefing that hospitalized persons are generally unaware of these problems, suggesting that some nurses may not involve hospitalized persons in the prevention of adverse events associated with nursing care. As this justification conflicts with patient’s right to be informed about their health status and is inconsistent with the perspectives of the WHO [78] and the Organization for Economic Co-operation and Development (OECD) [184], regarding the importance of patient involvement in safer and more person-centered care, the research team decided to retain these items.

### 4.2. Field Testing of the Final Version of the MISSCARE Survey-Patient-PT

#### 4.2.1. Characteristics of Field-Test Participants

A total of 141 hospitalized persons were invited to participate in the study, of whom 139 agreed to participate. A total of 135 people returned the survey, corresponding to a response rate of 97.13%. A worsening clinical condition was the main reason for non-return. All returned questionnaires contained complete and clear answers to all items in the MISSCARE Survey-Patient-PT. Most of the questionnaires were completed by the participants.

The sample included 79 women (58.5%) and 56 men (41.5%), of whom 104 had completed primary or secondary education (77.04%), 91 were mainly aged between 55 and 84 (67.40%), and of these, only 10 reported having no previous hospitalizations (7.4%). 

Regarding nurse-sensitive adverse events, 40 participants (29.6%) reported that their IV drip stopped because it was completely empty, 24 (17.8%) reported swelling of the hand or arm due to the IV, 16 (11.9%) reported acquiring an infection during their hospital stay, and 15 (11.1%) reported a skin lesion or pressure ulcer. Seven participants (5.2%) reported medication administration errors, and two (1.5%) reported falls. Sample characteristics are presented in Table 1.

The sample in this study is also characterized by experiencing moderate performance of several nursing activities: (a) being informed at each shift about the nurse responsible for providing care; (b) discussing the care plan with the nurse; (c) receiving information in advance about tests and/or procedures; (d) being listened to when expressing doubts or concerns about care or illness; (e) sharing opinions or ideas regarding their care; (f) being supervised by nurses when performing oral hygiene; (g) being supervised by nurses when getting out of bed to sit in a chair; and (h) being supervised by nurses when walking around the ward. Participants also reported short response times to machine or monitor alarms, call bell activation, receiving assistance after using the call bell, and obtaining help to go to the bathroom. High levels of supervision during bathing were also reported. Table 2 presents descriptive statistics for the items of the MISSCARE Survey-Patient-PT.

#### 4.2.2. Construct Validity 

Construct Validity of the MISSCARE Survey-Patient-PT was assessed using EFA. Data suitability was confirmed by a KMO value of 0.721 and a statistically significant Bartlett’s sphericity test [χ^2^(78) = 932.890, *p* < 0.001]. The final EFA solution retained the same number of items and dimensions as the original instrument [71] (Table 3) and explained 66.7% of the total variance, exceeding the recommended threshold for Social Sciences research [185]. Since the items loaded on the same factors as in the original instrument [71], the original factor labels were maintained. 

The Time to Response dimension included items tr_1, ”When a monitor or other machine sounded the alarm, how long, on average, did it take nurses to respond?” (Quando um monitor ou outra máquina tocava o alarme, quanto tempo, em média, levaram os enfermeiros a dar resposta?); tr_2, ”When you rang the bedside bell, how long did it take, on average, to get a response?” (Quando tocava à campainha de chamada, quanto tempo levou, em média, a ter uma resposta?); tr_3, “After your call was answered, how long did it take, on average, to receive the help you requested?” (Depois do pedido de chamada ser atendido, quanto tempo levou, em média, a receber a ajuda que pediu?); and tr_4, “When you needed help going to the bathroom, on average, how long did it take for help to arrive?” (Quando precisou de ajuda para ir à casa de banho, em média, quanto tempo levou a chegar ajuda?). The Time to Response was defined as the extent to which nursing care is provided to a patient with a minimal waiting time [186] and in a manner that does not compromise Patient Safety [187].

In turn, the Communication dimension comprised items co_1, “On average, how often were you informed during each shift about who was the nurse responsible for providing your care?” (Em média, com que frequência foi informado(a) em cada turno sobre quem era o(a) enfermeiro(a) responsável pela prestação dos seus cuidados?); co_2, “On average, how often did nurses discuss your care plan with you?” (Em média, com que frequência os enfermeiros discutiram consigo o seu plano de cuidados?); co_3, “On average, how often did nurses provide you with information about the tests and/or procedures you underwent during your hospital stay, namely when they would be performed and what they consisted of, etc.?” (Em média, com que frequência os enfermeiros lhe deram informações sobre os exames e/ou procedimentos que realizou durante o internamento, nomeadamente, o momento em que seriam feitos e no que consistiam, etc.?”); co_4, “On average, how often did nurses listen to you when you had questions or concerns about your care or illness?” (Em média, com que frequência os enfermeiros o(a) escutaram quando surgiram dúvidas ou preocupações sobre os seus cuidados ou doença?); and co_5, “On average, how often did nurses consider your opinions and ideas about what should be done regarding your care?” (Em média, com que frequência os enfermeiros consideraram as suas opiniões e ideias sobre o que devia ser feito relativamente aos seus cuidados?). Communication was defined as an interpersonal interaction in which the nurse focuses on the patient’s needs to promote a safe exchange of information [188].

Finally, the following items were included in the Basic Care dimension: bc_1, “On average, how often did nurses ensure that your teeth were brushed and/or your mouth was washed (or did they provide this care when you were unable to do so yourself)?” (Em média, com que frequência os enfermeiros se certificaram de que os seus dentes foram escovados e/ou que a boca foi lavada, ou lhe prestaram esses cuidados quando não foi capaz de o fazer?”; bc_2, “On average, how often did nurses ensure that you bathed or kept yourself clean during your hospital stay?” (Em média, com que frequência os enfermeiros se certificaram de que tomava banho ou se mantinha limpo(a) durante o internamento?); bc_3, “On average, how often did the nurses assist you or ensure that you got out of bed and sat in a chair?” (Em média, com que frequência os enfermeiros o(a) ajudaram ou se certificaram de que se levantava da cama e que se sentava num cadeirão?); and bc_4, “On average, how often did the nurses assist you or ensure that you were walking?” (Em média, com que frequência os enfermeiros o(a) ajudaram ou se certificaram de que caminhava?). In this study, Basic Care was defined as those aspects of care that can be performed under the supervision of nurses [189], which are essential to safety, regardless of the clinical situation, cultural background, or healthcare context [190].

**Figure 3:**
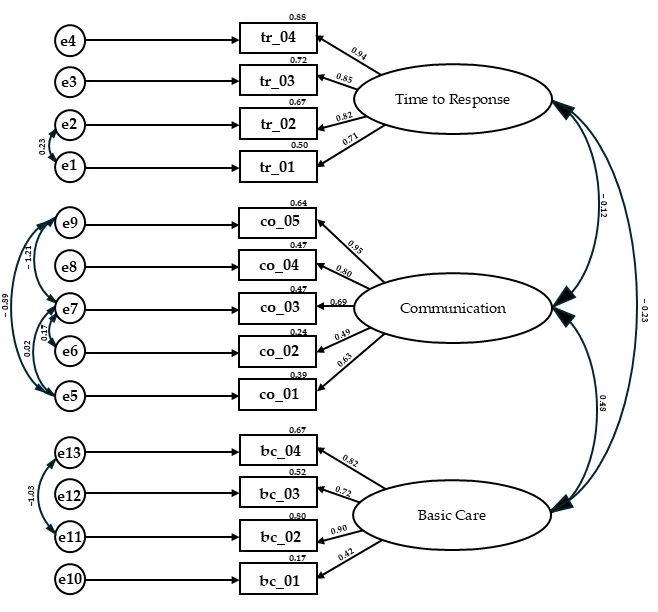
Final model of confirmatory factor analysis for the MISSCARE Survey-Patient-PT.

CFA initially indicated an inadequate fit for the three-factor model in the Portuguese sample (n = 135), primarily due to a χ^2^/df value > 3, which indicates discrepancies between the observed and model-implied covariance matrices [149]. The initial fit indices were as follows: X^2^(62) = 198,596; *p* < 0.001, χ^2^/df = 3203; RMSEA = 0.128 (90% CI: 0.108–0.148, PCLOSE < 0.001); PCFI = 0.673 and CFI = 0.847. Subsequently, model fit was improved by correlating error terms between conceptually related items: tr_2 with tr_1 (Time to Response); co_5 with co_3, co_5 with co_1, co_3 with co_2, and co_3 with co_1 (Communication); and bc_4 with bc_2 (Basic Care). These modifications yielded improved fit and provided support for the Structural Validity of the instrument. The revised model showed better fit indices: X^2^(56) = 52,421, *p* < 0.001, χ^2^/df = 2722; RMSEA = 0.113 (90% CI: 0.092–0.135, PCLOSE < 0.001); PCFI = 0.640 and CFI = 0.892; with acceptable values of χ^2^/df = 2722 and PCFI (*p* = 0.001) and a higher CFI value (0.892 versus 0.870), although the RMSEA remained above the recommended cutoff values. According to the guidelines proposed by Hair et al. [149], evidence of adequate model fit should be based on the chi-square statistic and its associated degrees of freedom, complemented by at least one incremental fit index and one absolute fit index, rather than requiring any specific absolute fit index, such as the RMSEA. Although widely used, the RMSEA has recognized limitations and may overestimate model misfit in small degrees of freedom; therefore, it should be interpreted alongside other model fit indices rather than in isolation [140]. Figure 3 shows the final model of the confirmatory factor analysis for the assessment instrument.

#### 4.2.3. Reliability 

For the overall instrument, Cronbach’s α was 0.818, indicating very good Internal Consistency. For the individual dimensions, Cronbach’s α values ranged from 0.766 to 0.902. Regarding the Mean Inter-Item Correlations (MIICs), the dimensions presented values between 0.460 and 0.700, showing good homogeneity among items. For the total overall instrument, the MIIC was 0.253. Finally, the mean item-total correlation values were >0.20 for both the dimensions and the overall instrument, which are acceptable values according to Kline [165]. According to DeVellis [191], an average inter-item correlation of 0.253 is considered adequate for assessment instruments with a Cronbach’s α of 0.80, provided they are multidimensional. Good Measurement Error characteristics were also observed for the final version of the MISSCARE Survey-Patient-PT and its dimensions, confirmed by SDC values < Minimal Important Change (MIC). Table 4 presents the Cronbach’s α, MIIC, and ITC values for both the dimensions and the overall instrument, while Table 5 shows the SEM, SDC, and MIC values.

#### 4.2.4. Convergent Validity

To assess Convergent Validity, the following hypothesis were formulated: the MISSCARE Survey-Patient-PT demonstrates Convergent Validity with patient-reported satisfaction with nursing care. To this end, a Pearson correlation analysis was performed to assess the relationship between the total score of the final version of the instrument and the mean response to the satisfaction question. Preliminary analysis indicated that the data were normally distributed according to the Kolmogorov–Smirnov test (*p* < 0.50).

The results showed a moderate and negative correlation between the two measures (r = −0.677; *p* < 0.001). This finding supports the Convergent Validity of the MISSCARE Survey-Patient-PT, as correlations greater than 0.50 are generally considered evidence of acceptable Convergent Validity (i.e., r > 0.50 [173]).

#### 4.2.5. Responsiveness

For the final version of the MISSCARE Survey-Patient-PT, the MIC exceeded the SDC by seven points for the total score, indicating that Measurement Error is smaller than the threshold for a meaningful change and supporting the instrument’s ability to detect important changes over time. Similarly, for each of the three dimensions, the MIC exceeded the SDC, indicating that each dimension is capable of detecting meaningful changes beyond the Measurement Error. Table 5 presents these values accompanied by descriptive statistics for both the dimensions and the overall instrument.

### 4.3. Assessment of Measurement Characteristics

#### 4.3.1. Interpretability 

For the overall instrument, floor (1.5%) and ceiling (8.1%) effects were within acceptable limits (<15%), indicating excellent discriminatory ability. However, the Response Time dimension showed a substantial ceiling effect (37%). The floor and ceiling effect values for each dimension and for the overall instrument are shown in Table 6.

#### 4.3.2. Feasibility 

All participants completed the MISSCARE Survey-Patient-PT without missing responses. The researcher responsible for data collection reported no difficulties in administering the questionnaire or in participants’ understanding of the items. The average time required to complete the survey was approximately five minutes.

## 5. Discussion

The objective of this study was to translate, adapt, and cross-culturally validate the MISSCARE Patient Survey [71] for the Portuguese population and to analyze the quality of its measurement properties assessed within the scope of this study.

### 5.1. Assessment of the Measurement Properties of the MISSCARE Survey-Patient-PT

This study addressed the challenge identified by Palese [192], who emphasized the need to prioritize comprehensive psychometric rigor and the evaluation of multiple measurement properties in the cross-cultural validation processes of MISSCARE instruments.

#### 5.1.1. Content Validity

Evidence for Content Validity includes Sampling Validity and Item Validity [193]. Sampling Validity refers to the quality of the sampling of the total content being tested, while Item Validity focuses on the relevance of the individual items in measuring the intended construct [194]. 

The very good agreement indices obtained for the clarity of the items in the pre-final version of the MISSCARE Survey-Patient-PT (S-CVI/Med = 0.98, S-CVI/UA = 0.84) support the Sampling Validity of the instrument. Item Validity is demonstrated by the excellent agreement indices regarding the relevance of the items included in the instrument (S-CVI/Med = 1.00, S-CVI/UA = 1.00). These results are consistent with the CVI/med and CVI/UA values reported by Si et al. [107], in the Chinese version of the instrument. 

The following factors contributed greatly to these results: (1) the involvement of two native Portuguese translators, one familiar with the MNC construct and the other without this background, who independently translated the original instrument into the target language; (2) two native English back-translators, both unfamiliar with the MNC construct; (3) the multidisciplinary committee, which included all translators, two coordinating nurses from the hospital where the validation study was conducted; (4) the application of the assessment instrument to a sample of hospitalized persons, followed by a cognitive debriefing; (5) the involvement of nurses from the target population as experts to assess the relevance of the items in the instrument; (6) the literature review on two adverse events and discussion with the NMs of the services where the data were collected; and (7) the inclusion of illustrative figures in the assessment instrument to facilitate patients’ understanding. Previous studies with PROMs have reported that the inclusion of illustrations in assessment instruments improves hospitalized persons’ understanding and motivation to answer questions [195,196]. 

In previous cross-cultural validation studies of the MISSCARE Patient Survey, Content Validity was assessed only through cognitive interviews [9] without the use of quantitative indices [9]. The empirical evidence of Content Validity obtained in this study supports both Conceptual Equivalence [197] and Semantic Equivalence [197] between the source and target versions of the instrument.

#### 5.1.2. Construct Validity

Sources of evidence for Construct Validity include the following: (1) evidence based on the content of the assessment instrument; (2) evidence based on response processes; and (3) evidence based on internal structure of the instrument [198]. 

Evidence based on the content of the instrument was demonstrated by the following: (1) the high proportion of explained variance obtained in the EFA model (66.7%) [185]; (2) the moderate correlation between the total score of the MISSCARE Survey-Patient-PT and the question aimed at assessing the quality of nursing care experienced by participants during hospitalization, which is acceptable [*r*(135) =−0.677; *p* < 0.001] [173]; and (3) the distribution of items across the same factors identified in the original instrument. The explained variance obtained in this study was higher than that reported in the Chinese validation study by Si et al. [107], by Albrechtsen et al. [199], and by Kalisch et al. [71]. In the study by Si et al. [107], a three-factor model with the same dimensions as the original instrument was also obtained: (1) Communication; (2) Basic Care; and (3) Time to Response.

Evidence based on response processes was demonstrated by appropriate MIIC coefficients and ITC amplitudes for the dimension Communication (MIIC = 0.469 and 0.498 ≤ ITC ≤ 0.686), the Basic Care dimension (MIIIC = 0.460 and 0.575 ≤ ITC ≤ 0.684), the Time to Response dimension (MIIC = 0.700 and 0.282 ≤ ITC ≤ 0.499), and for the overall instrument (MIIC and ITC) [162,165]. 

In turn, evidence based on the internal structure of the MISSCARE Survey-Patient-PT was demonstrated by the adequate Structural Validity indices obtained in the CFA model (X^2^(56) = 52.421, *p* < 0.001, χ^2^/df = 2.722; PCFI = 0.640 and CFI = 0.892) [149,151]. Construct Validity was also supported by an adequate sample size for performing the EFA and CFA (ratio of 10 participants per item) [124], confirmed by adequate KMO values (KMO = 0.721) and Bartlett’s statistically significant sphericity test [χ^2^(78) = 932.890, *p* < 0.001] [143,144]. However, the full sample was retained for both EFA and CFA, as splitting the dataset would have resulted in two subsamples of approximately 67 participants each, meaning that neither subsample would have met the COSMIN recommendation of at least 100 participants for the assessment of Structural Validity [200]. This approach is supported by Van Prooijen and Van der Kloot [201], who argue that, although the use of independent samples for exploratory and confirmatory factor analyses is preferable, using the same sample is acceptable when the exploratory analysis is conducted to verify an expected theoretical structure rather than to generate a new, data-driven factor model.

The sample used in this study was smaller than those used in other validation studies, including the Chinese version (n = 225) [107], the Turkish version (n = 267) [108], and the Danish version (n = 284) by Albrechtsen et al. [199]. 

The Construct Validity obtained for the MISSCARE Survey-Patient-PT reinforces both Conceptual Equivalence and Item Equivalence [197] between the source and target versions of the instrument.

The Communication dimension is consistent with previous studies [41,63] and reflects an area that requires nurses to develop Safe Communication skills [64]. Safe Communication is characterized by a set of activities involving the collection and sharing of information, clarification, and verification of the accuracy of interpretations, working collaboratively with clients, families, and other health professionals to achieve common goals related to the provision of safe, high-quality care [188]. It involves the transmission, collection, and exchange of sufficient information for shared understanding, as well as the transmission and correct interpretation of information, using communication with each other to validate the accuracy of the content of the messages communicated [202]. It also includes the interpretation of verbal messages in an unambiguous way, using interaction with others to reduce uncertainty or doubt, the framing of the interaction itself within local circumstances, such as time pressure or environmental noise that can create barriers to shared understanding, and responding verbally and non-verbally to expressed needs and expectations to maximize the likelihood of understanding [202]. According to Levett-Jones et al. [203], some examples of unsafe communication in nursing practice are as follows: (1) failure to give the patient the opportunity to ask questions; (2) failure to respond appropriately to these questions; (3) failure to respect a patient’s opinion (even if the patient’s opinion may be medically inaccurate, their observations are usually accurate and can be very valuable), and (4) inadequate or inaccurate advice on self-management and failure to communicate with other relevant healthcare professionals in order to provide a reasonable standard of care. To mitigate the occurrence of MNC, NMs should promote the enhancement of nurses’ safe communication skills [63].

The Basic Care dimension of MNC is consistent with previous studies [41,204] and reflects the importance of nurses having metacognitive Care Coordination skills to optimize the management of resources available for the care to be provided [205]. Care Coordination is a proactive approach that brings together professionals and caregivers to meet hospitalized persons’ needs and ensure that they receive integrated, person-centered care across different settings [206]. In nursing, this skill is highly dependent on nurses’ educational backgrounds [207]. Poor coordination can lead to duplicate care, care provided at the wrong time, unnecessary care, or no care at all [208]. In a study by Carthon et al. [209], which examined the relationship between MNC and hospital readmissions, it was concluded that for every 10-percentage-point increase in the proportion of nurses reporting MNC, the probability of patient readmission increased by 6%, partly due to deficiencies in care coordination. The care coordination process includes comprehensive assessments, care planning, patient and family teaching/training for self-management skills, coordination among healthcare providers, and proactive monitoring and evaluation, along with ongoing guidance and support [210]. To coordinate hospitalized persons’ clinical care, nurses need to make clinical decisions, set priorities, delegate safely, acquire organizational skills, utilize resources, manage time, and evaluate their hospitalized persons’ outcomes [211]. Care coordination is recognized as an appropriate model for acute care hospital settings [212]. Consequently, NMs should implement processes that strengthen nurses’ ability to coordinate patient care [213]. 

The Time to Response as a dimension of MNC is also consistent with some studies. To prevent the occurrence of MNC related to the Time to Response, nurses need to have metacognitive skills related to patient surveillance [214]. This is defined as “a process to primarily identify threats to patient health and safety through purposeful and ongoing acquisition, interpretation and synthesis of patient data for clinical decision making” [215]. It is also conceptualized as a collective effort of interventions performed over time by multiple nurses and those who depend on them functionally, as well as interventions performed individually [216]. Poor Patient Surveillance can make the difference between a good and a bad outcome at any single point in time [217]. Daniels [218] reported an increase in the high response time of the care team in relation to aspects related to personal hygiene and fall prevention as a result of frequent bedside rounds by nurses. The implementation of Nursing Bedside Handover (NBH) three times a day in the services that participated in this study may explain the high levels of Time to Response found (faster nursing response). In this type of bedside rounding, nurses can collect patient data through the following: (1) direct observation; (2) communication with hospitalized persons, family members, and team nurses; (3) review of nursing documentation systems; and (4) retrieval of relevant data through these systems. These data are interpreted and converted into synthesized information through cognitive processes (rational and intuitive), with the meaning of this information and the degree of risk of injury being assessed in relation to the trajectory of the patient’s clinical condition, leading to the planning of the appropriate course of action for the patient, not only in terms of interventions but also in terms of continuous monitoring [217]. Consequently, Patient Surveillance extends beyond simple observation and requires advanced clinical knowledge and training among nurses [219].

In a study conducted by Paiva et al. [220] the implementation of Nurse Case Management is advocated by nurses in a Portuguese hospital as a strategy to minimize the occurrence of MNC. This care model enables nurses to play a key role in ensuring safe communication, coordinating patient care, and monitoring hospitalized persons’ clinical conditions.

#### 5.1.3. Convergent Validity

Convergent Validity of the MISSCARE Survey-Patient-PT was supported by a correlation ≥ 0.50 [173] between the assessment instrument and the question assessing patient satisfaction with nursing care during hospitalization. The correlation coefficient obtained in this study was substantially stronger (r = −0.677; *p* < 0.001) than that reported by Kalisch et al. [71] (r = 0,25, *p* < 0.001). This evidence of Convergent Validity further supports the Construct Validity of the assessment instrument; however, it should be interpreted with caution, as Convergent Validity was assessed using a single global item assessing satisfaction with nursing care, consistent with the original validation study.

#### 5.1.4. Reliability

In this study, a Cronbach’s α coefficient similar to that of the original instrument was obtained (0.818 versus 0.860) [71], as well as coefficients comparable to those reported in previous cross-cultural adaptation studies [9,107,108]. Cronbach’s α coefficients for the dimensions were also obtained, ranging from acceptable to excellent. These values are consistent with the coefficients reported in previous cross-cultural adaptation studies [9,107,108]. In exploratory research within the Social Sciences, Cronbach’s α values between 0.60 and 0.70 are considered acceptable indicators of Internal Consistency [146]. However, Internal Consistency reflects only one aspect of Reliability. Although the present findings support the Internal Consistency of the MISSCARE Survey-Patient-PT, temporal stability was not evaluated through a Test–Retest design. Given the nature of the instrument, which assesses patients’ experiences of MNC during a specific hospitalization, the construct is inherently dynamic and cannot be assumed to remain stable throughout the hospital stay. Consequently, the construct stability required by the COSMIN methodology for the assessment of Test–Retest reliability [176] is difficult to ensure, as patients’ experiences continue to evolve until the hospitalization episode has ended.

#### 5.1.5. Responsiveness

To our knowledge, this is the first study reporting responsiveness-related indices for the MISSCARE Survey-Patient [71]. Although Responsiveness was not directly assessed using longitudinal data, evidence related to the instrument’s Potential Responsiveness was explored by comparing SDC and MIC. The MIC exceeded the SDC by seven points for the total instrument, indicating that important changes would be expected to exceed the Measurement Error and suggesting that the MISSCARE Survey-Patient-PT has the potential to detect meaningful change. However, the degree of responsiveness of an assessment instrument is largely determined by the context in which it is being used [221].

### 5.2. Practical Features of Using the Instrument

The Communication and Basic Care dimensions demonstrated good discriminative ability, whereas the Time to Response dimension showed a substantial ceiling effect. This ceiling effect was not reported in previous validation studies of the MISSCARE Survey-Patient [9,107,108] and may reflect relatively short response times by nurses in the services included in this study. One possible explanation for this finding relates to participants’ previous experiences of long response times in emergency departments prior to admission to the wards included in this study, as some participants reported during the individual debriefing. Another possible explanation may be the presence of adequate nurse staffing levels in the participating units. Nevertheless, the Time to Response dimension remains useful for monitoring the occurrence of MNC across different hospital units and services, where response times may vary depending on multiple contextual factors.

The existence of a ceiling effect alerts researchers to the need for a careful research design and the selection of measures appropriate to the research objectives [222]. In healthcare, it can even be useful for marketing purposes and to provide a sense of security to both healthcare professionals and healthcare organizations [223]. However, when a ceiling effect is identified within an assessment instrument, modifications should be considered to increase its discriminatory capacity [224]. Several strategies may be used to reduce ceiling effects, namely the following: (1) increasing the number of response options in the Likert scale; (2) using continuous rating scales such as Visual Analog Scales (VASs); (3) using unbalanced bidirectional/positively-packed scales; and (4) providing clear anchors for each response option to reduce ambiguity in interpretation [225]. Because patients perceive urgency differently depending on their health condition, the perceived value of nursing care may depend on how nurses respond to this sense of urgency [226]. Consequently, the ceiling effect identified in the Time to Response dimension can be mitigated by expanding the number of responses to 7 points, combined with anchors, such as the following: 1. Immediate (<2 min); 2. Very Short (2–5 min); 3. Short (5–10 min); 4. Acceptable (11–15 min); 5. Long (16–20 min); 6. Very Long (21–30 min); 7. Too Long (>30 min). A PREM designed to reduce information loss at the upper end of the scale can help healthcare organizations to more accurately assess the experience of hospitalized persons and the impact of change management interventions implemented [223]. This is particularly important for NMs, as the ability to design and implement change management interventions is a fundamental skill that should be developed during their academic training [227].

### 5.3. Study Strengths, Limitations, and Future Directions

The main strengths of this study include adherence to a methodological guideline throughout the translation and cross-cultural adaptation process and the comprehensive evaluation of the instrument’s measurement properties. Its main limitation is the recruitment of participants from a single hospital institution, which may limit the generalizability of the findings to other healthcare settings. Another limitation is that the EFA and CFA were initially conducted using the same dataset, consistent with the approach proposed by Van Prooijen and Van Der Kloot [201]. Nevertheless, the factorial structure should be confirmed in an independent dataset in future studies. Although Convergent Validity was evaluated using the same approach adopted in the original validation study [71], future studies should examine Convergent Validity using additional validated instruments measuring related constructs, such as patient experience, perceived quality of nursing care, or Patient Safety. Furthermore, future longitudinal studies are needed to formally establish the Responsiveness of the MISSCARE Survey-Patient-PT in accordance with COSMIN guidelines [228], as the present study inferred Responsiveness indirectly through the comparison between the MIC and the SDC, indicating that the instrument has the potential to detect meaningful change. Therefore, further research is needed to expand the evidence for the measurement properties of the MISSCARE Survey-Patient-PT. In particular, future studies should, also, assess Construct Validity through hypothesis testing, discriminant validity, measurement invariance, and differential item functioning (DIF), as well as examine the discriminatory capacity, predictive validity, and Test–Retest Reliability.

As further studies are warranted to evaluate the measurement properties of the MISSCARE Survey-Patient-PT in other healthcare settings before its use can be generalized beyond acute hospital care, future research should examine its applicability in contexts such as rehabilitation, long-term care, mental health, and primary healthcare. Furthermore, future studies should use this PREM in nursing intervention studies analyzing the perception of MNC and/or the safety of nursing care in different hospital contexts in Portugal. The instrument may also be used in international comparative studies, particularly with countries where cross-culturally adapted versions of the MISSCARE Survey-Patient are available. To maximize the accuracy of patient perceptions, it is recommended that future studies include a minimum hospitalization period of eight days as an inclusion criterion. Finally, future research should integrate this measurement instrument as an electronic Patient-Reported Experience Measure (ePREM) combined with predictive analytics, not only to facilitate its implementation but also to optimize the monitoring of nursing management interventions aimed at the safety of nursing care provision. 

## 6. Implications for Comprehensive Care and Health Services

Comprehensive Health Care is defined by the Australian Commission on Safety and Quality in Health Care (ACSQHC) as the coordinated provision of total health care aligned with the care goals and health care needs expressed by hospitalized persons, while also considering the impact of health problems on their lives and well-being [229]. Consequently, from the perspective of Comprehensive Health Care, the availability of a measurement instrument that allows us to understand hospitalized persons’ perceptions of the occurrence of Nurse Sensitive Adverse Events provides an opportunity to highlight nursing care processes that are often implicit and undervalued in health care settings. In addition, incorporating hospitalized persons’ voices on these care processes allows NMs to align nursing care provision with the needs and expectations of hospitalized persons and their families, particularly in relation to fundamental activities such as communication, basic care, and patient monitoring during hospitalization. At the health service level, the availability of a PREM for the MNC complements the data reported by Portuguese nurses with data provided by hospitalized persons, thereby strengthening the accountability and transparency of healthcare services. Finally, this study provides Portuguese NMs with a robust measurement instrument for monitoring the quality of nursing care, benchmarking, and continuous improvement. The instrument also supports the evaluation of care models that promote nurses’ skills in Safe Communication, Care Coordination, and Patient Surveillance, which are essential for preventing the occurrence of MNC.

## 7. Conclusions

The quality of a PREM is defined by the results obtained in the assessment of measurement properties [142]. The final version of MISSCARE Survey-Patient-PT (File S1) provides good validity and reliability for the ongoing monitoring of hospitalized persons’ perceptions of the occurrence of MNC in Portuguese acute health services. This instrument may support NMs in monitoring nursing care processes, particularly in nursing practice environments that promote the active participation of hospitalized persons in their own care. As Jones et al. [230] argue “ongoing monitoring and open collaboration can help minimize the risk of MNC during implementation of an innovation focused on improving quality and patient safety”.

## Figures and Tables

**Table 1:** Characteristics of the study sample.

Characteristic		*n*	%
Current hospitalization unit			
	Medical unit	63	46.7
	Surgical unit	72	53.3
Number of previous hospitalizations in the last 5 years			
	No hospitalizations	10	7.4
	1 to 3 hospitalizations	104	77.0
Gender			
	Female	79	58.5
	Male	56	41.5
Age			
	<44 years old	16	11.8
	45 to 54	16	11.9
	55 to 64	30	22.2
	65 to 74	24	17.8
	75 to 84	37	27.4
	≥85 years old	12	8.9
Education level			
	Less than 4 years of schooling	7	5.2
	Basic education	58	43.0
	Secondary education/high school diploma	46	32.1
	Higher education	18	13.3
	Postgraduate education	6	4.5
During this hospitalization, did you happen to have any falls?			
	Yes	2	1.5
	No	133	98.5
	Not sure	0	0.0
Did you suffer any skin lesions or pressure ulcers during this hospitalization?			
	Yes	15	11.1
	No	118	87.4
	Not sure	2	1.5
Did you contract any infections during your stay in hospital?			
	Yes	16	11.9
	No	104	77.0
	Not sure	15	11.1
During this hospitalization, were there any errors in the administration of your medication?			
	Yes	7	5.2
	No	110	81.5
	Not sure	18	13.3
During this hospitalization, did your IV drip stop because it was completely empty?			
	Yes	40	29.6
	No	88	65.2
	Not sure	7	5.2
During this hospitalization, did your hand or arm become swollen because of the IV?			
	Yes	24	17.8
	No	108	80.0
	Not sure	3	2.2

Note: *n* = 135 participants.

**Table 2:** Descriptive statistics for items of MISSCARE Survey-Patient-PT.

Items	*n*	Mean	SD
co_1: “On average, how often were you informed during each shift about who was the nurse responsible for providing your care?” (Em média, com que frequência foi informado(a) em cada turno sobre quem era o(a) enfermeiro(a) responsável pela prestação dos seus cuidados?)	135	3.03	1.50
co_2: “On average, how often did nurses discuss your care plan with you?” (Em média, com que frequência os enfermeiros discutiram consigo o seu plano de cuidados?)	135	2.76	1.30
co_3: “On average, how often did nurses provide you with information about the tests and/or procedures you underwent during your hospital stay, namely when they would be performed and what they consisted of, etc.?” (Em média, com que frequência os enfermeiros lhe deram informações sobre os exames e/ou procedimentos que realizou durante o internamento, nomeadamente, o momento em que seriam feitos e no que consistiam, etc.?)	135	3.62	1.32
co_4: “On average, how often did nurses listen to you when you had questions or concerns about your care or illness?” (Em média, com que frequência os enfermeiros o(a) escutaram quando surgiram dúvidas ou preocupações sobre os seus cuidados ou doença?)	135	3.64	1.60
co_5: “On average, how often did nurses consider your opinions and ideas about what should be done regarding your care?” (Em média, com que frequência os enfermeiros consideraram as suas opiniões e ideias sobre o que devia ser feito relativamente aos seus cuidados?)	135	3.09	1.58
bc_1: “On average, how often did nurses ensure that your teeth were brushed and/or your mouth was washed (or did they provide this care when you were unable to do so yourself)?” (Em média, com que frequência os enfermeiros se certificaram de que os seus dentes foram escovados e/ou que a boca foi lavada, ou lhe prestaram esses cuidados quando não foi capaz de o fazer?)	135	2.58	1.58
bc_2: “On average, how often did nurses ensure that you bathed or kept yourself clean during your hospital stay?” (Em média, com que frequência os enfermeiros se certificaram de que tomava banho ou se mantinha limpo(a) durante o internamento?)	135	4.15	1.30
bc_3: “On average, how often did the nurses assist you or ensure that you got out of bed and sat in a chair?” (Em média, com que frequência os enfermeiros o(a) ajudaram ou se certificaram de que se levantava da cama e que se sentava num cadeirão?)	135	3.53	1.50
bc_4: “On average, how often did the nurses assist you or ensure that you were walking?” (Em média, com que frequência os enfermeiros o(a) ajudaram ou se certificaram de que caminhava?)	135	3.05	1.53
tr_1:”When a monitor or other machine sounded the alarm, how long, on average, did it take nurses to respond?” (Quando um monitor ou outra máquina tocava o alarme, quanto tempo, em média, levaram os enfermeiros a dar resposta?)	135	4.48	0.80
tr_2:“When you rang the bedside bell, how long did it take, on average, to get a response?” (Quando tocava à campainha de chamada, quanto tempo levou, em média, a ter uma resposta?)	135	4.32	1.05
tr_3:”After your call was answered, how long did it take, on average, to receive the help you requested?” (Depois do pedido de chamada ser atendido, quanto tempo levou, em média, a receber a ajuda que pediu?)	135	4.22	0.95
tr_4: “When you needed help going to the bathroom, on average, how long did it take for help to arrive?” (Quando precisou de ajuda para ir à casa de banho, em média, quanto tempo levou a chegar ajuda?)	135	4.45	0.98

**Table 3:** Factor analysis of MISSCARE Survey-Patient-PT items.

Item	Components *			Communalities
	Time to Response	Communication	Basic Care	
tr_1	0.831	-	-	0.451
tr_2	0.876	-	-	0.567
tr_3	0.873	-	-	0.571
tr_4	0.925	-	-	0.763
co_1	-	0.626	-	0.597
co_2	-	0.749	-	0.432
co_3	-	0.729	-	0.771
co_4	-	0.857	-	0.787
co_5	-	0.733	-	0.594
bc_1	-	-	0.476	0.699
bc_2	-	-	0.869	0.781
bc_3	-	-	0.881	0.794
bc_4	-	-	0.652	0.860

* Loadings > 0.40.

**Table 4:** Internal Consistency coefficients for the dimensions and overall instrument of the final version of the MISSCARE Survey-Patient-PT (*n* = 135).

Dimensions	Cronbach’s α	Mean Inter-Item Correlations	Range of Item–Total Correlations
Communication	0.817	0.469	0.498–0.686
Basic Care	0.766	0.460	0.575–0.684
Time to Response	0.902	0.700	0.282–0.499
Overall Instrument	0.818	0.253	0.282–0.686

**Table 5:** Mean, standard deviation (SD), standard error measurement (SEM), Smallest Detectable Change (SDC), and Minimal Important Change (MIC) dimensions and overall instrument of the final version of the MISSCARE Survey-Patient-PT (*n* = 135).

Dimensions	Mean	SD	SEM	SDC	MIC
Communication	16.238	5.610	2.430	6.736	11.220
Basic Care	13.325	4.534	2.190	6.062	9.068
Time to Response	6.518	3.336	1.040	2.880	6.672
Instrument Total	36.081	8.904	3.790	10.50	17.808

**Table 6:** Ceiling and floor effects for dimensions of MISSCARE Survey-Patient-PT and for total instrument (*n* = 135).

Dimensions	Frequency of Minimum Score	Floor Effect (%)	Frequency of Maximum Score	Ceiling Effect (%)
Communication	2	1.5	16	11.9
Basic care	2	1.5	19	14.1
Time to Response	2	1.5	50	37.0
Total	2	1.5	11	8.1

## Data Availability

Data are contained within the article or Supplementary Materials.

## Supplementary Materials

The following supporting information can be downloaded at: https://www.mdpi.com/article/10.3390/nursrep16090312/s1, Table S1: The COSMIN reporting guideline for studies on measurement properties of PROMs Version 2.0 (Checklist) [94]; Table S2: STROBE Statement—Checklist of items that should be included in reports of cross-sectional studies [231]; Table S3: Comparative table of all versions of the MISSCARE Survey Patient-PT*; File S1: Questionário MISSCARE—Experiência da Pessoa Internada relativamente aos Cuidados de Enfermagem, Versão Portuguesa (MISSCARE Survey-Patient, Portuguese Version).

## Public Involvement Statement

Hospitalized persons participated in this study, specifically in the pre-test as experts and field test of the pre-final and final versions of the MISSCARE Survey-Patient-PT. Their insights informed revisions aimed at enhancing item clarity, phrasing accuracy, and contextual appropriateness. Nonetheless, hospitalized persons did not take part in designing the research, determining outcome measures, conducting data analyses, or interpreting findings. Following study completion, results will be shared with participants and the broader hospital community through institutional briefings and academic presentations, ensuring transparency and recognizing patient contributions. Hospitalized persons were not involved in preparing this manuscript.

## Guidelines and Standards Statement

This manuscript was drafted in accordance with COSMIN reporting guideline for studies on measurement properties of PROMs https://doi.org/10.1007/s11136-025-03950-x (accessed on 19 December 2025) and The Strengthening the Reporting of Observational Studies in Epidemiology (STROBE) Statement: Guidelines for reporting observational studies https://doi.org/10.1016/j.ijsu.2014.07.013 (accessed on 19 December 2025) to report this validation study.

## Abbreviations

The following abbreviations are used in this manuscript:

ACSQHC	Australian Commission on Safety and Quality in Health Care
Amos	Analysis of Moment Structures
AVE	Average Variance Extracted
CFA	Confirmatory Factor Analysis
CFI	Comparative Fit Index
COSMIN	COnsensus-based Standards for the selection of health Measurement INstruments
CVI	Content Validity Index
df	Degrees of freedom
EFA	Exploratory factor analysis
ePREM	Electronic Patient-Reported Experience Measure
I-CVI	Item-Content Validity Index
IV	Intravascular
ITC	Item-total Correlation
JCI	Joint Commission International
KMO	Kaiser-Meyer-Olkin
MIC	Minimal Important Change
MIIC	Mean Inter-Item Correlation
MNC	Missed Nursing Care
NBH	Nursing Bedside Handover
OECD	Organization for Economic Co-operation and Development
PCFI	Parsimony Comparative Fit Index
PNFI	Parsimony Normed Fit Index
PPP	Public-Private Partnership
PREMs	Patient-Reported Experience Measures
PROMs	Patient-Reported Outcome Measures
RMSEA	Root Mean Square Error of Approximation
S-CVI	Scale Content Validity Index
SEM	Standard Error of Measurement
SD	Standard Deviation
SDC	Smallest Detectable Change
SDG	Sustainable Development Goal
SPSS	Statistical Package for the Social Sciences
SRM	Standardized Response Mean
STROBE	Strengthening the Reporting of Observational Studies in Epidemiology
UA	Universal Agreement
USA	Unite States of America
VAS	Visual Analog Scales
WHO	World Health Organization

## Appendix A

### Appendix A.1

**Table A1:** Summary of the pre-test of the clarity of items in the pre-final version of the MISSECARE Survey-Patient (*n* = 10).

Item	Not Clear	Clear	I-CVI	UA	S-CVI/Med	S-CVI/UA
co_1: “On average, how often were you informed during each shift about who was the nurse responsible for providing your care?” (Em média, com que frequência foi informado(a) em cada turno sobre quem era o(a) enfermeiro(a) responsável pela prestação dos seus cuidados?)	0.00	10.00	1.00	1.00	0.98	0.84
co_2: “On average, how often did nurses discuss your treatment with you?” (Em média, com que frequência os enfermeiros discutiram consigo o seu tratamento?	1.00	9.00	0.90	0.00		
co_3: “On average, how often did nurses give you information about tests (e.g., X-rays, MRI, CT scan) and/or procedures you underwent during your hospitalization (namely, when they would be performed and what they consisted of, etc.)?” [Em média, com que frequência os enfermeiros lhe deram informações sobre os exames (por exemplo, raio-X, ressonância magnética, TAC) e/ou procedimentos que realizou durante o internamento (nomeadamente, o momento em que seriam feitos e no que consistiam, etc.)?]	1.00	9.00	0.90	0.00		
co_4: “On average, how often did nurses listen to you when you had questions or concerns about your care or illness?”(Em média, com que frequência os enfermeiros o(a) escutaram quando surgiram dúvidas ou preocupações sobre os seus cuidados ou doença?)	0.00	10.00	1.00	1.00		
co_5: “On average, how often did nurses consider your opinions and ideas about what should be done regarding your care?” (Em média, com que frequência os enfermeiros consideraram as suas opiniões e ideias sobre o que devia ser feito relativamente aos seus cuidados?)	0.00	10.00	1.00	1.00		
bc_1: “On average, how often did nurses ensure that your teeth were brushed and/or your mouth was washed (or did they provide this care when you were unable to do so yourself)?” (Em média, com que frequência os enfermeiros se certificaram de que os seus dentes foram escovados e/ou que a boca foi lavada, ou lhe prestaram esses cuidados quando não foi capaz de o fazer?)	0.00	10.00	1.00	1.00		
bc_2: “On average, how often did nurses ensure that you bathed or kept yourself clean during your hospital stay?” (Em média, com que frequência os enfermeiros se certificaram de que tomava banho ou se mantinha limpo(a) durante o internamento?)	0.00	10.00	1.00	1.00		
bc_3: “On average, how often did the nurses assist you or ensure that you got out of bed and sat in a chair?” (Em média, com que frequência os enfermeiros o(a) ajudaram ou se certificaram de que se levantava da cama e que se sentava num cadeirão?)	0.00	10.00	1.00	1.00		
bc_4: “On average, how often did the nurses assist you or ensure that you were walking?” (Em média, com que frequência os enfermeiros o(a) ajudaram ou se certificaram de que caminhava?)	0.00	10.00	1.00	1.00		
tr_1: ”When a monitor or other machine sounded the alarm, how long, on average, did it take nurses to respond” (Quando um monitor ou outra máquina tocava o alarme, quanto tempo, em média, levaram os enfermeiros a dar resposta?)	0.00	10.00	1.00	1.00		
tr_2: “When you rang the bedside bell, how long did it take, on average, to get a response?” (Quando tocava à campainha de chamada, quanto tempo levou, em média, a ter uma resposta?)	0.00	10.00	1.00	1.00		
tr_3: ”After your call was answered, how long did it take, on average, to receive the help you requested?” (Depois do pedido de chamada ser atendido, quanto tempo levou, em média, a receber a ajuda que pediu?)	0.00	10.00	1.00	1.00		
tr_4: “When you needed help going to the bathroom, on average, how long did it take for help to arrive?” (Quando precisou de ajuda para ir à casa de banho, em média, quanto tempo levou a chegar ajuda?)	0.00	10.00	1.00	1.00		

Note: CVI—Content validity index of items. UA—Universal agreement indices, where (1) means 100% agreement and (0) means < 100% agreement. S-CVI/Med—Median of S-CVI indices. CVI/UA—Median of UA indices.

### Appendix A.2

**Table A2:** Summary of the pre-test of relevance of items in the pre-final version of the MISSECARE Survey-Patient (n = 10).

Item	Not Relevant	Relevant	I-CVI	UA	S-CVI/Med	S-CVI/UA
co_1: “On average, how often were you informed during each shift about who was the nurse responsible for providing your care?” (Em média, com que frequência foi informado(a) em cada turno sobre quem era o(a) enfermeiro(a) responsável pela prestação dos seus cuidados?)	0.00	10.00	1.00	1.00	1.00	1.00
co_2: “On average, how often did nurses discuss your care plan with you?” (Em média, com que frequência os enfermeiros discutiram consigo o seu plano de cuidados?)	0.00	10.00	1.00	1.00		
co_3: “On average, how often did nurses provide you with information about the tests and/or procedures you underwent during your hospitalization (namely, when they would be performed and what they consisted of, etc.)? [Em média, com que frequência os enfermeiros lhe forneceram informações sobre os exames e/ou procedimentos que realizou durante o seu internamento (ou seja, quando seriam realizados e em que consistiam, etc.)?]	0.00	10.00	1.00	1.00		
co_4: “On average, how often did nurses listen to you when you had questions or concerns about your care or illness?” (Em média, com que frequência os enfermeiros o(a) escutaram quando surgiram dúvidas ou preocupações sobre os seus cuidados ou doença?)	0.00	10.00	1.00	1.00		
co_5: “On average, how often did nurses consider your opinions and ideas about what should be done regarding your care?” (Em média, com que frequência os enfermeiros consideraram as suas opiniões e ideias sobre o que devia ser feito relativamente aos seus cuidados?)	0.00	10.00	1.00	1.00		
bc_1: “On average, how often did nurses ensure that your teeth were brushed and/or your mouth was washed (or did they provide this care when you were unable to do so yourself)?” (Em média, com que frequência os enfermeiros se certificaram de que os seus dentes foram escovados e/ou que a boca foi lavada, ou lhe prestaram esses cuidados quando não foi capaz de o fazer?)	0.00	10.00	1.00	1.00		
bc_2: “On average, how often did nurses ensure that you bathed or kept yourself clean during your hospital stay?” (Em média, com que frequência os enfermeiros se certificaram de que tomava banho ou se mantinha limpo(a) durante o internamento?)	0.00	10.00	1.00	1.00		
bc_3: “On average, how often did the nurses assist you or ensure that you got out of bed and sat in a chair?” (Em média, com que frequência os enfermeiros o(a) ajudaram ou se certificaram de que se levantava da cama e que se sentava num cadeirão?)	0.00	10.00	1.00	1.00		
bc_4: “On average, how often did the nurses assist you or ensure that you were walking?” (Em média, com que frequência os enfermeiros o(a) ajudaram ou se certificaram de que caminhava?)	0.00	10.00	1.00	1.00		
tr_1: ”When a monitor or other machine sounded the alarm, how long, on average, did it take nurses to respond” (Quando um monitor ou outra máquina tocava o alarme, quanto tempo, em média, levaram os enfermeiros a dar resposta?)	0.00	10.00	1.00	1.00		
tr_2: “When you rang the bedside bell, how long did it take, on average, to get a response?” (Quando tocava à campainha de chamada, quanto tempo levou, em média, a ter uma resposta?)	0.00	10.00	1.00	1.00		
tr_3: ”After your call was answered, how long did it take, on average, to receive the help you requested?” (Depois do pedido de chamada ser atendido, quanto tempo levou, em média, a receber a ajuda que pediu?)	0.00	10.00	1.00	1.00		
tr_4: “When you needed help going to the bathroom, on average, how long did it take for help to arrive?” (Quando precisou de ajuda para ir à casa de banho, em média, quanto tempo levou a chegar ajuda?)	0.00	10.00	1.00	1.00		

Note: CVI—Content validity index of items. UA—Universal agreement indices, where (1) means 100% agreement and (0) means < 100% agreement. S-CVI/Med—Median of S-CVI indices. CVI/UA—Median of UA indices.
