# Supplementary material for: Portuguese Version of the MISSCARE Survey-Patient: A Cross-Cultural Validation Study
Source: Nurs Rep. 2026 Sep 1;16(9):312. doi: 10.3390/nursrep16090312 (PMC13609928; doi:10.3390/nursrep16090312)
Supplement: Supplementary file 1 [file nursrep-16-00312-s001.zip › Survey_S4_v3.pdf]

Questionário MISSCARE – Experiência da Pessoa Internada relativamente aos Cuidados de Enfermagem, Versão Portuguesa

(MISSCARE Survey-Patient, Portuguese Version)

Instrução *(Intruction)*:

Responda a cada uma das questões, assinalando com um X a resposta correta ou preenchendo o espaço fornecido. Não existem respostas certas ou repostas erradas. A informação que fornecer será mantida confidencial (*Answer each question by marking the correct answer with an X or filling in the space provided. There are no right or wrong answers. The information you provide will be kept confidential).*

Parte I – Atividades de cuidados de enfermagem

(Part I - Nursing care activities)

(co_1) Em média, com que frequência foi informado(a) em cada turno sobre quem era o(a) enfermeiro(a) responsável pela prestação dos seus cuidados? (*On average, how often were you informed during each shift about who was the nurse responsible for providing your care?*)

1)_____Nunca *(Never)*
2)_____Raramente *(Rarely)*
3)_____Às vezes *(Sometimes)*
4)_____Geralmente *(Usually)*
5)_____Sempre *(Always)*

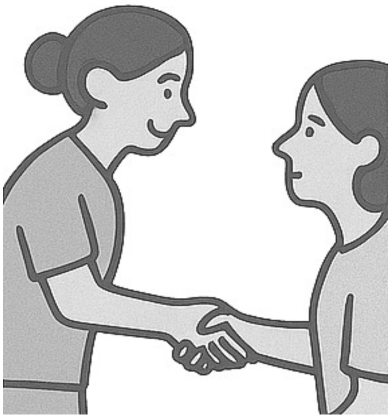

OpenAI (2025)

(co_2) Em média, com que frequência os enfermeiros discutiram consigo o seu plano de cuidados? *(On average, how often did nurses discuss your care plan with you?)*

1)_____Nunca *(Never)*
2)_____Raramente *(Rarely)*
3)_____Às vezes *(Sometimes)*
4)_____Geralmente *(Usually)*
5)_____Sempre *(Always)*

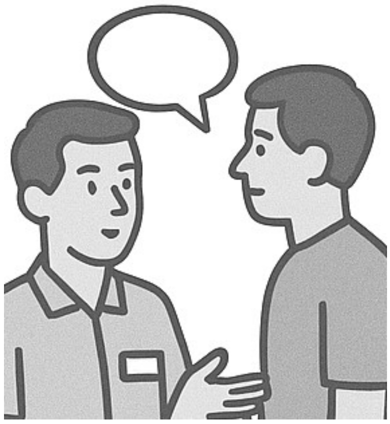

OpenAI (2025)

(co_3) Em média, com que frequência os enfermeiros lhe deram informações sobre os exames ou procedimentos que teve de realizar (nomeadamente, o momento em que foram agendados, no que consistiram, etc.)? *[On average, how often did nurses provide you with information about the tests and/or procedures you underwent during your hospitalization (namely, when they would be performed and what they consisted of, etc.)?]*

1)_____Nunca *(Never)*
2)_____Raramente *(Rarely)*
3)_____Às vezes *(Sometimes)*
4)_____Geralmente *(Usually)*
5)_____Sempre *(Always)*

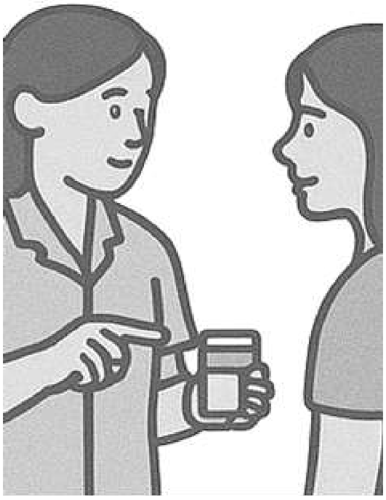

OpenAI (2025)

(co_4) Em média, com que frequência os enfermeiros o(a) escutaram quando surgiram dúvidas ou preocupações sobre os seus cuidados ou doença? *(On average, how often did nurses listen to you when you had questions or concerns about your care or illness?)*

1)_____Nunca *(Never)*
2)_____Raramente *(Rarely)*
3)_____Às vezes *(Sometimes)*
4)_____Geralmente *(Usually)*
5)_____Sempre *(Always)*

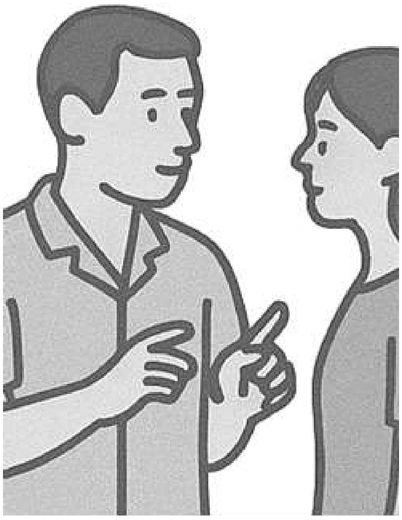

OpenAI (2025)

(co_5) Em média, com que frequência os enfermeiros consideraram as suas opiniões e ideias sobre o que devia ser feito relativamente aos seus cuidados? *[On average, how often did nurses consider your opinions and ideas about what should be done regarding your care?]*

1)_____Nunca *(Never)*
2)_____Raramente *(Rarely)*
3)_____Às vezes *(Sometimes)*
4)_____Geralmente *(Usually)*
5)_____Sempre *(Always)*

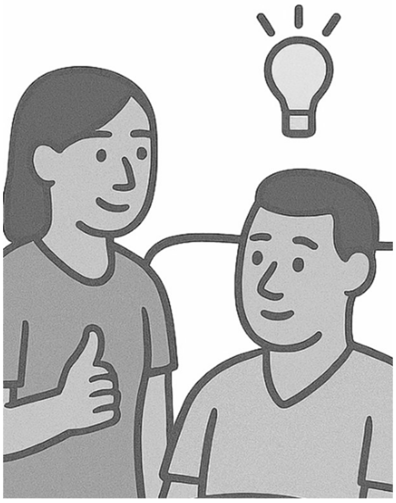

OpenAI (2025)

(bc_1) Em média, com que frequência os enfermeiros se certificaram de que os seus dentes foram escovados e/ou que a boca foi lavada (ou lhe prestaram esses cuidados quando não foi capaz de o fazer)? *[On average, how often did nurses ensure that your teeth were brushed and/or your mouth was washed (or did they provide this care when you were unable to do so yourself)?]*

1)_____**Nunca** *(Never)*
2)_____**Raramente** *(Rarely)*
3)_____**Às vezes** *(Sometimes)*
4)_____**Geralmente** *(Usually)*
5)_____**Sempre** *(Always)*

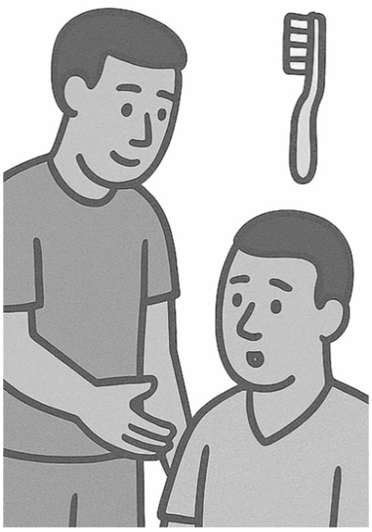

OpenAI (2025)

(bc_2) Em média, com que frequência os enfermeiros se certificaram de que tomava banho ou se mantinha limpo(a) durante o internamento? *(On average, how often did nurses ensure that you bathed or kept yourself clean during your hospital stay?)*

1)_____**Nunca** *(Never)*
2)_____**Raramente** *(Rarely)*
3)_____**Às vezes** *(Sometimes)*
4)_____**Geralmente** *(Usually)*
5)_____**Sempre** *(Always)*

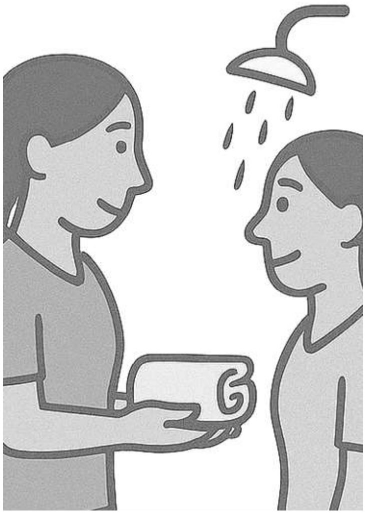

OpenAI (2025)

(bc_3) Em média, com que frequência os enfermeiros o(a) ajudaram ou se certificaram de que se levantava da cama e que se sentava num cadeirão? *(On average, how often did the nurses assist you or ensure that you got out of bed and sat in a chair?)*

1)_____**Nunca** *(Never)*
2)_____**Raramente** *(Rarely)*
3)_____**Às vezes** *(Sometimes)*
4)_____**Geralmente** *(Usually)*
5)_____**Sempre** *(Always)*

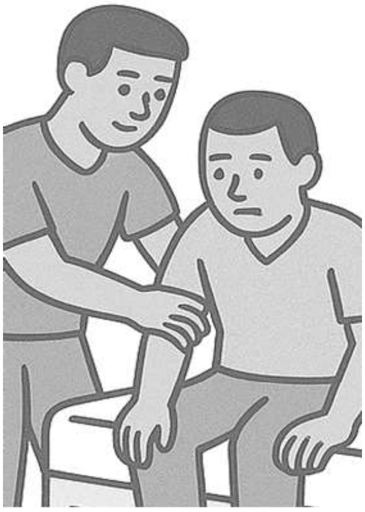

OpenAI (2025

(bc_4) Em média, com que frequência os enfermeiros o(a) ajudaram ou se certificaram de que caminhava? *(On average, how often did the nurses assist you or ensure that you were walking?)*

1)_____**Nunca** *(Never)*
2)_____**Raramente** *(Rarely)*
3)_____**Às vezes** *(Sometimes)*
4)_____**Geralmente** *(Usually)*
5)_____**Sempre** *(Always)*

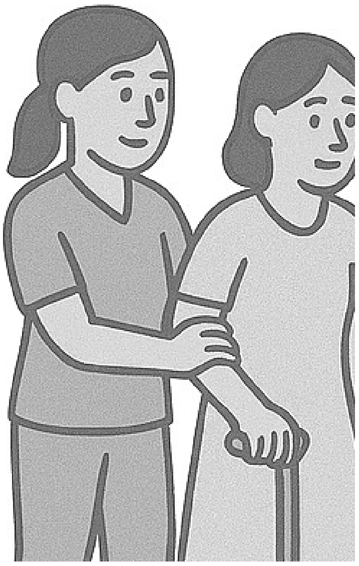

OpenAI (2025)

(tr_1) Quando um monitor ou outra máquina tocava o alarme, quanto tempo, em média, levaram os enfermeiros a dar resposta? *(When a monitor or other machine sounded the alarm, how long, on average, did it take nurses to respond?)* R

1)_____**Menos do que 5 minutos** *(Less than 5 minutes)*
2)_____**5 a 10 minutos** *(5 to 10 minutes)*
3)_____**11 a 20 minutos** *(11 to 20 minutes)*
4)_____**21 a 30 minutos** *(21 to 30 minutes)*
5)_____**Mais do que 30 minutos** *(More than 30 minutes)*

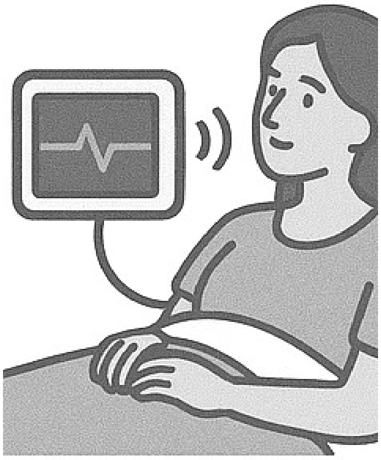

OpenAI (2025)

(tr_2) Quando tocava à campainha de chamada, quanto tempo levou, em média, a ter uma resposta? *(When you rang the bedside bell, how long did it take, on average, to get a response?)* R

1)_____**Menos do que 5 minutos** *(Less than 5 minutes)*
2)_____**5 a 10 minutos** *(5 to 10 minutes)*
3)_____**11 a 20 minutos** *(11 to 20 minutes)*
4)_____**21 a 30 minutos** *(21 to 30 minutes)*
5)_____**Mais do que 30 minutos** *(More than 30 minutes)*

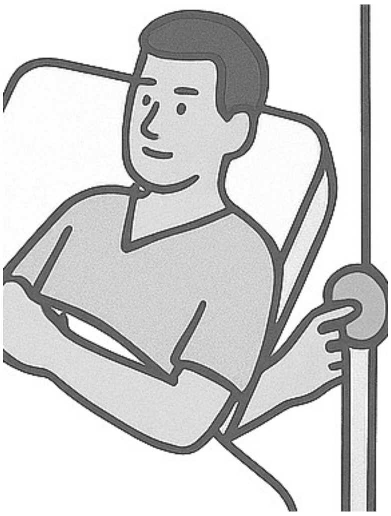

OpenAI (2025)

R: Reverse coded

(tr_3) Depois do pedido de chamada ser atendido, quanto tempo levou, em média, a receber a ajuda que pediu? *(After your call was answered, how long did it take, on average, to receive the help you requested?)* R

1)_____Menos do que 5 minutos *(Less than 5 minutes)*
2)_____5 a 10 minutos *(5 to 10 minutes)*
3)_____11 a 20 minutos *(11 to 20 minutes)*
4)_____21 a 30 minutos *(21 to 30 minutes)*
5)_____Mais do que 30 minutos *(More than 30 minutes)*

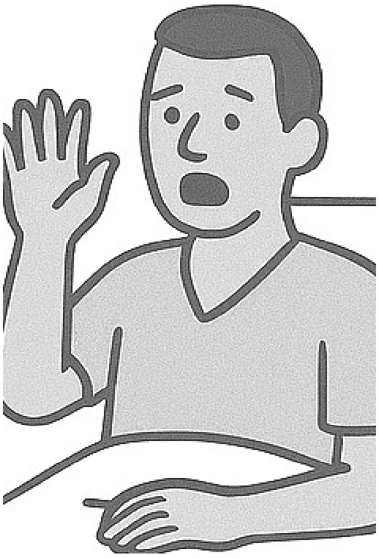

OpenAI (2025)

(tr_4) Quando precisou de ajuda para ir à casa de banho, em média, quanto tempo levou a chegar ajuda? *(When you needed help going to the bathroom, on average, how long did it take for help to arrive?)* R

1)_____Menos do que 5 minutos *(Less than 5 minutes)*
2)_____5 a 10 minutos *(5 to 10 minutes)*
3)_____11 a 20 minutos *(11 to 20 minutes)*
4)_____21 a 30 minutos *(21 to 30 minutes)*
5)_____Mais do que 30 minutos *(More than 30 minutes)*

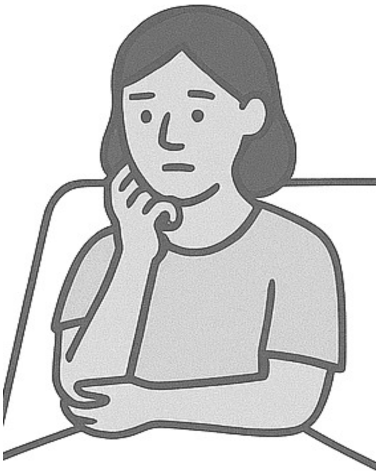

OpenAI (2025)

Parte II – Eventos adversos sensíveis aos cuidados de enfermagem
(Part II - Adverse events sensitive to nursing care)

(ae_1) Neste internamento aconteceu-lhe ter alguma queda? *(During this hospitalization, did you happen to have any falls?)*

1)_____Sim *(Yes)*
2)_____Não *(No)*
3)_____Não sei *(Not sure)*

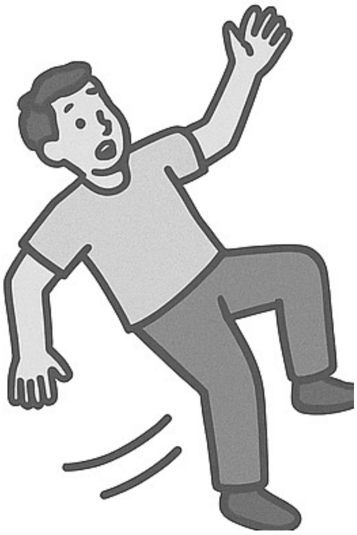

OpenAI (2025)

R: Reverse coded

(ae_2) Neste internamento aconteceu-lhe ficar com alguma ferida na pele ou úlcera por pressão? *(Did you suffer any skin lesions or pressure ulcers during this hospitalization?)*

1)_____**Sim** *(Yes)*
2)_____**Não** *(No)*
3)_____**Não sei** *(Not sure)*

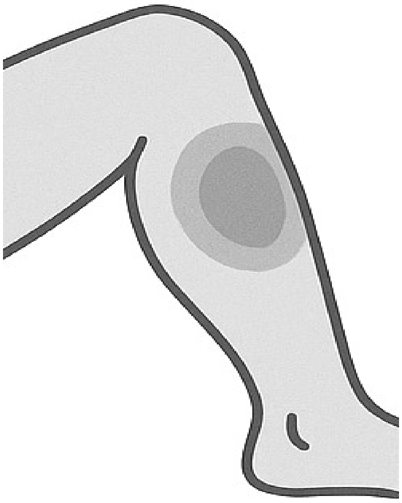

OpenAI (2025)

(ae_3) Neste internamento aconteceu-lhe ter alguma infeção adquirida durante a permanência no hospital? *(Did you contract any infections during your stay in hospital?)*

1)_____**Sim** *(Yes)*
2)_____**Não** *(No)*
3)_____**Não sei** *(Not sure)*

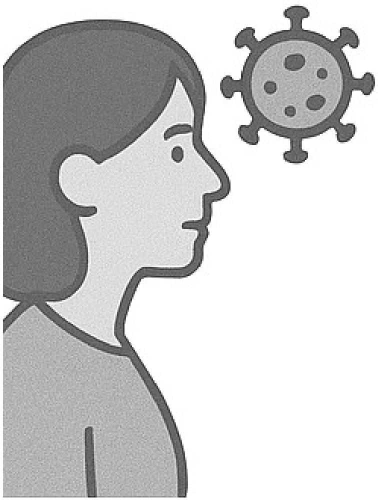

OpenAI (2025)

(ae_4) Neste internamento aconteceu-lhe algum erro na administração da sua medicação? *(During this hospitalization, were there any errors in the administration of your medication?)*

1)_____**Sim** *(Yes)*
2)_____**Não** *(No)*
3)_____**Não sei** *(Not sure)*

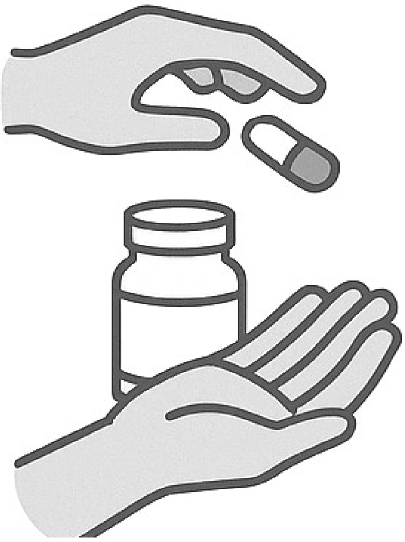

OpenAI (2025)

(ae_5) Neste internamento aconteceu-lhe ficar com o soro sem pingar por estar completamente vazio? *(During this hospitalization, did your IV drip stop because it was completely empty?)*

1)_____**Sim** *(Yes)*
2)_____**Não** *(No)*
3)_____**Não sei** *(Not sure)*

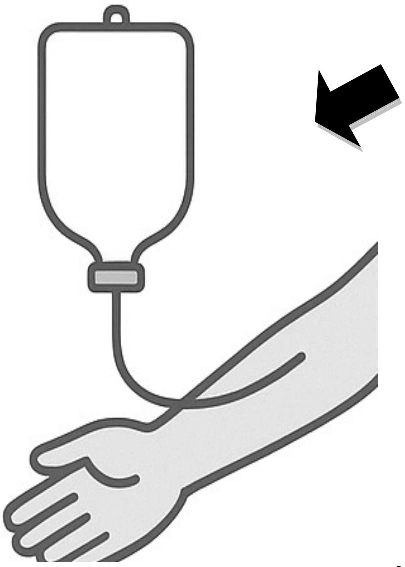

OpenAI (2025)

(ae_6) Neste internamento aconteceu-lhe ficar com a mão ou braço inchados por causa do soro? *(During this hospitalization, did your hand or arm become swollen because of the IV?)*

1)_____**Sim** *(Yes)*
2)_____**Não** *(No)*
3)_____**Não sei** *(Not sure)*

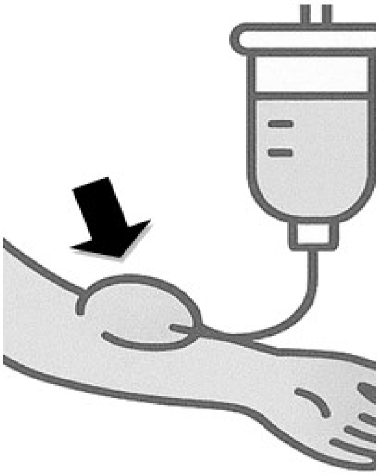

OpenAI (2025)

(sat) De um modo geral, como classifica os cuidados de enfermagem que lhe foram prestados durante este internamento? *(Overall, how would you rate the nursing care you received during this hospital stay?)*

1)_____**Péssimos** *(Bad)*
2)_____**Fracos** *(Poor)*
3)_____**Razoáveis** *(Fair)*
4)_____**Muito bons** *(Very Good)*
5)_____**Excelentes** *(Excellent)*

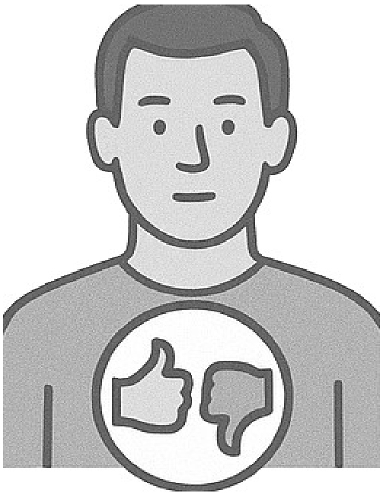

OpenAI (2025)

Parte III – Dados Sociodemográficos
(Part III - Sociodemographic data)

Género *(Gender)*:

1	2	3
**Feminino** *(Female)*	**Masculino** *(Male)*	**Outro** *(Other)*

Idade (*Age*):

1	**Até 24 anos** *(Up to 24 years old)*
2	**Entre os 25 e os 34** *(Between 25 and 34)*
3	**Entre os 35 e os 44** *(Between 35 and 44)*
4	**Entre os 45 e os 54** *(Between 45 and 54)*
5	**Entre os 55 e os 64** *(Between 55 and 64)*
6	**Entre os 65 e os 74** *(Between 65 and 74)*
7	**Entre os 75 e os 84** *(Between 75 and 84)*
8	**85 ou mais anos** *(85 or older)*

Habilitações literárias *(Academic qualifications)*:

1	**Menos de 4 anos de escolaridade** *(Less than 4 years of schooling)*
2	**1º ciclo do ensino básico (4ª classe)** *[1st cycle of basic education (4th grade)]*
3	**2º ciclo do ensino básico (5º e 6º ano)** *[2nd cycle of basic education (5th and 6th grades)]*
4	**3º ciclo do ensino básico (7º ao 9º ano)** *[3rd cycle of basic education (7th to 9th grades)]*
5	**Ensino secundário (10º ao 12º ano)** *[Secondary education (10th to 12th grades)]*
6	**Curso técnico-profissional** *(Vocational/technical education)*
7	**Bacharelato** *[Bachelor's degree (short-cycle)]*
8	**Licenciatura** *(Bachelor's degree)*
9	**Mestrado** *(Master's degree)*
10	**Doutoramento** *[Doctoral degree (PhD)]*

Serviço em que está internado/a *(Unit where you are hospitalized)*:

____

-Já esteve internado/a anteriormente? *(Have you ever been hospitalized before?)*

1	2
**Sim** *(Yes)*	**Não** *(No)*

-Quantas internamentos teve anteriormente nos últimos 5 anos? *(How many hospitalizations have you had in the past 5 years?)*

__________ vezes *(times)*

- Durante o seu internamento, esteve acompanhado(a) por uma pessoa significativa para si (companheiro(a), cônjuge, pessoa amiga, etc.)? *(During your hospital stay, were you accompanied by a significant person in your life (partner, spouse, friend, etc.)?*

1	2
**Sim** *(Yes)*	**Não** *(No)*

R: Reverse coded

Neste internamento aconteceu-lhe ficar com outros problemas? Se sim, indique-o(s), por favor *(Did you experience any other problems during this hospital stay? If so, please list them).*

Obrigado pela sua participação! *(Thank you for your participation!)*
