# Supplementary material for: Portuguese Version of the MISSCARE Survey-Patient: A Cross-Cultural Validation Study
Source: Nurs Rep. 2026 Sep 1;16(9):312. doi: 10.3390/nursrep16090312 (PMC13609928; doi:10.3390/nursrep16090312)
Supplement: Supplementary file 1 [file nursrep-16-00312-s001.zip › Table S3_Comparative table.pdf]

**Table S3.** Comparative table of all versions of the MISSCARE Survey Patient-PT*.

| Original items | Translation<br>Translator 1 | Translation<br>Translator 2 | Translation committee** | Back translation<br>Translator 4 | Back translation<br>Translator 5 | Multidisciplinary Committee *** |
|---|---|---|---|---|---|---|
| **A - Items from the MISSCARE Survey-Patient** | | | | | | |
| **co _1.**How often were you clear about which specific nurse was assigned to take care of you for the shift? | **co_1.**Com que frequência foi informado sobre o enfermeiro que estava encarregado de cuidar de si durante o turno? | **co_1.**Com que frequência foi informado sobre quem é o/a enfermeiro/a que estava responsável pela prestação dos seus cuidados durante o turno? | **co_1**.Em média, com que frequência, foi informado(a) sobre que enfermeiro(a) estaria responsável pela prestação dos seus cuidados em cada turno? | **co _1.**On average, how often, were you informed about which nurse would be responsible for the provision of the care on each shift? | **co _1.**On average, how often were you told about which nurse would be responsible for the provision of your care on each shift? | **co _1**.Em média, com que frequência foi informado(a) em cada turno sobre quem era o(a) enfermeiro(a) responsável pela prestação dos seus cuidados? |
| **co _2.**How often did your nursing staff discuss your treatment with you? | **co _2.**Com que frequência a equipa de enfermagem discutiu o seu tratamento consigo? | **co _2.**Com que frequência é que a equipa de enfermagem discutiu consigo o seu tratamento? | **co _**2.Em média, com que frequência, a equipa de enfermagem discutiu consigo o seu tratamento? | **co _2.**On average, how often, did the nursing team discuss your treatment with you? | **co _2.**On average, how often did the nursing team discuss your treatment with you? | **co _2.**Em média, com que frequência os enfermeiros discutiram consigo o seu tratamento? |
| **co _3.**How often did your nursing staff give you information about | **co _3.**Com que frequência é que a equipa de enfermagem lhe | **co _3.**Com que frequência a equipa de enfermagem lhe | **co _3.**Em média, com que frequência, a equipa de enfermagem lhe | **co _3.**On average, how often, did the nursing team provide you with | **co _3.**On average, how often did the nursing team give you information about the | **co _3**.Em média, com que frequência os enfermeiros lhe deram informações |

| Original items | Translation Translator 1 | Translator 2 | Translation committee** | Back translation Translator 4 | Translator 5 | Multidisciplinary Committee *** |
|---|---|---|---|---|---|---|
| tests (e.g. x-ray, MRI, CT scan) and/or procedures you received during this hospitalization (timing, what would be involved, etc.)? | deu informações sobre os exames médicos (por exemplo, raio-X, ressonância magnética, tomografia computorizada) e/ou os procedimentos que recebeu durante este internamento (timing, o que estaria envolvido, etc.)? | forneceu informações sobre os exames (por exemplo, raio-X, ressonância magnética, tomografia computorizada) e/ou procedimentos que realizou durante o internamento (prazos, o que estaria envolvido, etc.)? | forneceu informações sobre os exames (por exemplo, raio-X, ressonância magnética, TAC) e/ou procedimentos realizados durante o internamento (nomeadamente, o momento em que seriam feitos e no que consistiam, etc.)? | information about the exams (for example, X-ray,magnetic resonance, CAT) and/or procedures performed during hospitalisation (namely, the moment at which they would be done and what they would involve, etc.)? | tests (for example, X-ray, MRI, CT) and/or procedures performed during your admission (namely, when they would be done and what they consisted of, etc)? | sobre os exames (por exemplo, raio-X, ressonância magnética, TAC) e/ou procedimentos que realizou durante o internamento (nomeadamente, o momento em que seriam feitos e no que consistiam, etc.)? |
| **co _4.**When you had a question or concern about your care or illness, did your nursing staff listen to you? | **co _4.**Quando teve uma pergunta ou preocupação sobre os seus cuidados ou doença, a equipa de enfermagem ouviu-o? | **co _4.**Quando tinha uma pergunta ou preocupação sobre os seus cuidados ou doença, a equipa de enfermagem ouvia-o? | **co _4.**Em média, com que frequência a equipa de enfermagem o(a) escutou quando surgiram dúvidas ou preocupações sobre os seus cuidados ou doença? | **co _4.** On average, how often did the nursing team listen to you when doubts or concerns arose about your care or disease? | **cm _4**. On average, how often did the nursing team listen to you when doubts or worries about your care or illness arose? | **co _4.**Em média, com que frequência os enfermeiros o(a) escutaram quando surgiram dúvidas ou preocupações sobre os seus |

| Original items | Translation<br>Translator 1 | Translator 2 | Translation committee** | Back translation<br>Translator 4 | Translator 5 | Multidisciplinary Committee *** |
|---|---|---|---|---|---|---|
| | | | | | | cuidados ou doença? |
| **co_5.**When you had an opinion about what needed to be done relative to your care, did the nursing staff consider your opinions and ideas? | **co _5.**Quando tinha uma opinião sobre o que precisava de ser feito relativamente aos seus cuidados, a equipa de enfermagem teve/ levou em conta as suas opiniões e ideias? | **co _5.**Quando tinha uma opinião sobre o que devia ser feito, relativamente aos seus cuidados, a equipa de enfermagem teve em conta as suas opiniões e ideias? | **co _**5.Em média, com que frequência a equipa de enfermagem considerou as suas opiniões e ideias sobre o que devia ser feito relativamente aos seus cuidados? | **co _5.**On average, how often did the nursing team take into account your opinions and ideas about what should be done with regard to your care? | **co _5.**On average, how often did the nursing team consider your opinions and ideas about what should be done regarding your care? | **co _5.**Em média, com que frequência os enfermeiros consideraram as suas opiniões e ideias sobre o que devia ser feito relativamente aos seus cuidados? |
| **bc_1.**How often did the nursing staff check with you to make sure your teeth were brushed and mouth rinsed (or provide the care if you could not do it yourself)? | **bc_1.**Com que frequência é que a equipa de enfermagem se certificava de que os seus dentes eram escovados e a boca lavada (ou prestavam esses cuidados se não o pudesse fazer)? | **bc_1.**Com que frequência é que a equipa de enfermagem se certificava de que a sua higiene oral era realizada (ou prestava esses cuidados se não o pudesse fazer)? | **bc_1.**Em média, com que frequência a equipa de enfermagem se certificou de que os seus dentes eram escovados e/ou que a boca era lavada (ou prestou esses cuidados caso não o pudesse fazer)? | **bc_1.**On average, how often did the nursing team check that your teeth were brushed and/or that your mouth was rinsed (or provided this care if you could not do it)? | **6 bc_1**On average, how often did the nursing team check that your teeth were brushed and/or that your mouth was washed (or provided this care if you couldn't do it)? | **bc_1.**Em média, com que frequência os enfermeiros se certificaram de que os seus dentes foram escovados e/ou que a boca foi lavada (ou lhe prestaram esses cuidados quando |

| Original items | Translation Translator 1 | Translator 2 | Translation committee** | Back translation Translator 4 | Translator 5 | Multidisciplinary Committee *** |
|---|---|---|---|---|---|---|
| | | | | | | não foi capaz de o fazer)? |
| **bc_2.**How often did the nursing staff check with you to make sure you had a bath or were kept clean throughout your hospitalization? | **bc_2.**Com que frequência é que a equipa de enfermagem se certificou de que tomava banho ou se mantinha limpo durante o seu internamento? | **bc_2.**Com que frequência é que a equipa de enfermagem se certificou de que tomava banho ou se mantinha limpo durante o internamento? | **bc_2.**Em média, com que frequência a equipa de enfermagem se certificou de que tomava banho ou se mantinha limpo(a) durante o internamento? | **bc_2.**On average, how often did the nursing team check that you had showered or you remained clean during the hospitalisation? | **bc_2.**On average, how often did the nursing team check that you had a shower or kept yourself clean during your admission? | **bc_2.**Em média, com que frequência os enfermeiros se certificaram de que tomava banho ou se mantinha limpo(a) durante o internamento? |
| **bc_3.**On average, how often did the nursing staff help you or monitor that you got out of bed and sat in a chair? | **bc_3.**Em média, com que frequência é que a equipa de enfermagem o ajudou ou monitorizou para que se levantasse da cama e se sentasse numa cadeira? | **bc_3.**Em média, com que frequência a equipa de enfermagem o ajudou ou certificou-se que se levantava da cama e se sentava numa cadeira? | **bc_3.**Em média, com que frequência a equipa de enfermagem o(a) ajudou ou se certificou de que se levantava da cama e que se sentava numa cadeira? | **bc_3.**On average, how often did the nursing team help you or check that you got up from the bed and that you sat on a chair? | **bc_3.**On average, how often did the nursing team help you or check that you got out of bed and that you sat in a chair? | **bc_3**.Em média, com que frequência os enfermeiros o(a) ajudaram ou se certificaram de que se levantava da cama e que se sentava num cadeirão? |

| Original items | Translation Translator 1 | Translator 2 | Translation committee** | Back translation Translator 4 | Translator 5 | Multidisciplinary Committee *** |
|---|---|---|---|---|---|---|
| **bc_4.**On average, how often did the nursing staff help you or monitor that you walked? | **bc_4.**Em média, com que frequência a equipa de enfermagem o ajudou ou monitorizou a sua mobilidade? | **bc_4.**Em média, com que frequência a equipa de enfermagem o ajudou ou monitorizou a andar? | **bc_4.**Em média, com que frequência a equipa de enfermagem o(a) ajudou ou se certificou de que caminhava? | **bc_4.**On average, how often did the nursing team help you or checked that you were walking? | **bc_4.**On average, how often did the nursing team help you or check that you walked? | **bc_4.**Em média, com que frequência os enfermeiros o(a) ajudaram ou se certificaram de que caminhava? |
| **tr_1.**When a monitor or other machine beeped, how long did it usually take the nursing staff to respond? | **tr_1.**Quando um monitor ou outra máquina emitia um sinal sonoro, quanto tempo demorava normalmente a equipa de enfermagem a responder? | **tr_1.**Quando um monitor ou outra máquina apitava, quanto tempo demorava normalmente a equipa de enfermagem a responder? | **tr_1.**Quando um monitor ou outra máquina soou o alarme, quanto tempo, em média, demorou a equipa de enfermagem a responder? | **tr_1.**When a monitor or other machine set off the alarm, how long, on average, did it take the nursing team to respond? | **tr_1.**When a monitor or other machine sounded the alarm, how long, on average, did the nursing team take to respond? | **tr_1.**Quando um monitor ou outra máquina tocava o alarme, quanto tempo, em média, levaram os enfermeiros a dar resposta? |
| **tr_2.**When you pushed your call light, how long on average did it take the nursing staff to answer? | **tr_2.**Quando acionou a luz de chamada, quanto tempo, em média, demorou a equipa | **tr_2.**Quando tocava a campainha de chamada, quanto tempo, em média, demorou a equipa | **tr_2.**Quando tocava à campainha de chamada, quanto tempo demorou, em média, a equipa de | **tr_2.**When you rang the call bell, how long did it take, on average, the nursing team to respond? | **tr_2.**When you pressed the call bell, how long, on average, did the nursing team take to respond? | **tr_2.**Quando tocava à campainha de chamada, quanto tempo levou, em média, a ter uma resposta? |

| Original items | Translation Translator 1 | Translator 2 | Translation committee** | Back translation Translator 4 | Translator 5 | Multidisciplinary Committee *** |
|---|---|---|---|---|---|---|
| | de enfermagem a responder? | de enfermagem a responder? | enfermagem a responder? | | | |
| **tr_3.**Once your call light was answered, how long on average did it take for you to receive the help you requested? | **tr_3.**Quando a sua luz de chamada foi atendida, quanto tempo demorou, em média, a receber a ajuda que tinha pedido? | **tr_3.**Quando tocava a campainha de chamada, quanto tempo demorou, em média, a receber a ajuda que pediu? | **tr_3.**Depois de o seu pedido de chamada ter sido atendido, quanto tempo demorou, em média, a receber a ajuda que pediu? | **tr_3.**After your call was answered, how long did it take, on average, to receive the help that you requested? | **tr_3.**After your call request was answered, how long did it take, on average, to receive the help you asked for? | **tr_3.**Depois do pedido de chamada ser atendido, quanto tempo levou, em média, a receber a ajuda que pediu? |
| **tr_4.**If you needed help to go to the bathroom, how long did it take the nursing staff to get into your room to help you? | **tr_4.**Se precisou de ajuda para ir à casa de banho, quanto tempo demorou a equipa de enfermagem a chegar ao seu quarto para o ajudar? | **tr_4.**Se precisou de ajuda para ir à casa de banho, quanto tempo demorou a equipa de enfermagem a entrar no seu quarto para o ajudar? | **tr_4.**Quando precisou de ajuda para ir à casa de banho, quanto tempo demorou, em média, a equipa de enfermagem a chegar ao seu quarto para o(a) ajudar? | **tr_4.**When you needed help to go to the bathroom, how long did it take, on average, the nursing team to arrive at your room to help you? | **tr_4.**When you needed help to go to the bathroom, how long did it take, on average, for the nursing team to arrive at your room to help you? | **tr_4.**Quando precisou de ajuda para ir à casa de banho, em média, quanto tempo levou a chegar ajuda? |
| **B - Questions included in the Sociodemographic Data Section** | | | | | | |
| **ae.**Did you experience any of the following | **ae.**Teve algum dos seguintes | **ae.**Teve algum dos seguintes problemas | **ae.**Teve algum dos seguintes problemas | **ae.**Did you have any of the following | **ae.**Did you have any of the following | **ae.**Neste internamento aconteceu-lhe |

| Original items | Translation | | Translation committee** | Back translation | | Multidisciplinary Committee *** |
|---|---|---|---|---|---|---|
| | Translator 1 | Translator 2 | | Translator 4 | Translator 5 | |
| problems during this hospitalization?<br>a)Fall<br>b)Skin breakdown / Pressure ulcer<br>c)Medication Administration Error<br>d)New Infection<br>e)IV running dry<br>f)IV leaking into your skin<br>g) other problems | problemas durante este internamento?<br>a)Quebra<br>b)Rutura da pele / Úlcera de pressão<br>c)Erro na administração de medicamentos<br>d)Nova infeção<br>f)IV a esgotar-se<br>g)Fuga de IV para a pele<br>g) outros problemas | durante esta hospitalização?<br>a) Queda<br>b) Alteração da integridade cutânea / Úlcera por pressão<br>c) Erro na administração terapêutica<br>d) Diagnóstico de uma nova infeção<br>e) Cateter intravenoso não funcional<br>f) Fuga de soro extra cateter intravenoso<br>g) outros problemas | durante este internamento?<br>a) Queda;<br>b) Lesão na pele / Úlcera por pressão;<br>c) Erro na administração da medicação;<br>d) infeção adquirida no internamento;<br>e) Soro a terminar sem ser substituído;<br>f) Infiltração do soro para a pele<br>g) outros problemas | problems during this hospitalisation?a) Fall;<br>b) Skin lesion / Pressure ulcer;<br>c) Error in administration of the medication;<br>d) Hospitalisation-acquired infection;<br>e) Saline ending without being replaced;<br>f) Saline infiltration into the skin<br>g) other problems | problems during this admission?<br>a) Fall;<br>b) Skin wound / Pressure ulcer;<br>c) Error in the administration of medication;<br>d) infection acquired in the admission;<br>e) Saline running out without being replaced;<br>f) Infiltration of saline into the skin<br>g) other problems | algum dos seguintes problemas:<br>a)Queda?<br>b) Lesão na pele ou úlcera por pressão?<br>c) Erro na administração de medicação?<br>d)Infeção adquirida no internamento?<br>f) Embalagen(s) de soro e/ou medicação vazia(s) sem ser removida(s)?<br>g)Soro a sair fora da veia e ir para a pele?<br>h)Outros problemas? |
| **sat.**Overall, how would you rate your nursing care while you were a | **sat.**De um modo geral, como classificaria os cuidados de enfermagem que | **sat.**Se precisou de ajuda para ir à casa de banho, quanto tempo demorou a equipa | **sat.**De um modo geral, como classificaria os cuidados de enfermagem que lhe | **sat.**Overall, how would you rate the nursing care that was provided | **sat.**In general, how would you rate the nursing care that you | **sat.**De um modo geral, como classifica os cuidados de enfermagem que |

| Original items | Translation | | Translation committee** | Back translation | | Multidisciplinary Committee *** |
|---|---|---|---|---|---|---|
| | Translator 1 | Translator 2 | | Translator 4 | Translator 5 | |
| patient during this hospitalization? | lhe foram prestados durante este internamento? | de enfermagem a entrar no seu quarto para o ajudar? | foram prestados durante este internamento? | to you during this hospitalisation? | were given during this admission? | lhe foram prestados durante este internamento? |

**Note:** *Adapted from Kalisch, B.J.; Xie, B.; Dabney, B.W. Patient-Reported Missed Nursing Care Correlated with Adverse Events. *Am. J. Med. Qual.* **2014**, *29*, 415–422, doi:10.1177/1062860613501715. © 2014 *Wolters Kluwer Health*, Inc; ** The translation committee included three translators (1, 2, and 3); ** The translation committee included three translators (1, 2, and 3); *** Participated in the Multidisciplinary Committee two coordinating nurse managers and two nurses from the host institution, one monolingual nurse without experience in the Missed Nursing Care, the translator 1, 2, 3, 4, and 5, and two members from the research team (PC and GT).
